# Topochemical Analysis of Pine Bark toward Structure‐First Biorefineries

**DOI:** 10.1002/cssc.71220

**Published:** 2026-11-12

**Authors:** Rustam Ibadov, Teemu Tomberg, Muhammad Awais, Idil Ertem, Joni Parkkonen, Paavo Penttilä, Monika Österberg, Bruno D. Mattos

**Affiliations:** ^1^ Department of Bioproducts and Biosystems School of Chemical Engineering Aalto University Espoo Finland; ^2^ Division of Pharmaceutical Chemistry and Technology Faculty of Pharmacy University of Helsinki Helsinki Finland; ^3^ Department of Physics Nanoscience Center University of Jyväskylä Jyväskylä Finland

**Keywords:** bark, biomass, biorefinery, lignocellulose, structure–activity relationships

## Abstract

Pine bark is an abundant, yet underutilized forestry by‐product often burnt for energy, despite its rich chemical functionality. Its broader use is limited by poor processability and high heterogeneity of products obtained through conventional fractionation. Most processes employed for bark have been traditionally designed for wood, a vastly different lignocellulosic precursor. In this work, we moved beyond bulk analysis by employing a topochemical approach to understand the bark recalcitrance and structural integrity. By advanced spectroscopic imaging combined with controlled solvent treatments and macroscale integrity analysis, we provided new insights into how bark architecture governs its recalcitrance in the context of biorefineries. Bark recalcitrance largely stems from the thick outer cell walls, dominated by conjugated C═C double bonds and unsaturated carbonyl structures, that are disrupted by alkaline agents. Removal of lipophilic extractives increases the fibers' cohesion, potentially requiring more energy input for fiber liberation. Furthermore, bark structure is loosened by removing pectins and phenolics in the middle lamella and cell corners. Macroscale texture depends on the nature, not just the amount, of removed components. By understanding the roles of each component in the integrity of the bark ultrastructure, we put forward a generic structure‐first concept to develop novel bark biorefineries.

## Graphical abstract

Moving beyond bulk with topochemical analysis: bark cellular structure revealed by stimulated Raman scattering(SRS) imaging, where two synchronized pump and Stokes laser beams drive molecular vibrations. Topochemical analysis by advanced spectroscopic imaging combined with controlled treatments and the monitoring of the macroscale integrity unravels new insights into how bark architecture governs its recalcitrance.

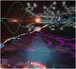

**Abbreviations**
BM4D: 4D block‐matching
BSTFA: N,O‐Bis(trimethylsilyl)trifluoroacetamide
CLS: classical least squares
DMF: *N*,*N*‐dimethylformamide
GC: gas chromatography
GPC: gel permeation chromatography
HPLC: high‐performance liquid chromatography
MS: mass spectrometry
PCA: principal component analysis
SRS: stimulated Raman scattering

## 1 Introduction

The shift toward renewable resources and a circular bioeconomy while avoiding competition with food systems is in increasing demand. Forest‐based biomass is uniquely positioned to supply large volumes of bioresources for developing sustainable materials through the higher‐value use of side streams from pulp, paper, and sawmill operations [1, 2]. Among these side streams, pine bark is available at large scales and remains underutilized, often burned for heat and energy rather than valorized into advanced materials. Bark functions as a protective barrier against physical damage, fire, insects, pathogens, and fungi; reduces water loss; thermally insulates the tree; and stores sugars, starch, and nutrients [3, 4, 5, 6]. Chemically, pine bark is rich in lignin, phenolic (e.g., condensed tannins, stilbenes, and lignans), and lipophilic (e.g., resin acids, fatty acids, and sterols) extractives, alongside cellulose, hemicellulose, and pectin, offering a wide selection of compounds with various intrinsic functionalities [7, 8, 9, 10, 11].

Despite decades of research on bark valorization, it is still approached through the lens of wood science, where bark is oversimplified into just another lignocellulosic material. Traditional bark refining strategies have overlooked its distinct anatomical, chemical, and functional nature. The traditional methods developed for wood refining do not transfer very well to bark, given their remarkably different microstructures and chemical composition. While wood is chemically dominated by carbohydrates, bark is generically defined by a substrate comprising mostly lignin and extractives cross‐linked within the carbohydrate polymer matrix. The extractives are commonly referred to as a wide range of small molecules, labeled as nonstructural elements, and have been largely approached as impurities. This is a general concept imported from wood, where extractives are minor structure‐disrupting components. In bark, however, extractives are abundant, and they carry multifunctionality within the bark tissue, including cohesion; thus, they should be viewed as key players for deconstructing and eventually valorizing bark. The higher lignin content and thus an increased number of covalent bonds between lignin and carbohydrates, in addition to extractives, have been labeled as making the material recalcitrant, but the origin and nature of this so‐called recalcitrance have been vastly overlooked. Characterization has been done to a high extent in bulk [11, 12], but once the material is ground and dissolved in a sequence of solvents for a full chemical composition analysis, a whole dimension of data related to the spatial distribution of each component is lost. The topochemical distribution of bark components at a cellular level prospectively holds the key to successfully unlocking the full potential of this inherently multifunctional material.

In this study, we integrate a multiscale investigation of bark tissues alongside their response to the selective removal of structural and so‐called non‐structural components to establish a concept of a structure‐first biorefinery. Selective removal of bark components is followed by an analysis of its microstructure and cohesion of the tissue, which is largely tethered to several biorefinery strategies, including fiber liberation, fractionation, and pulping. We utilized the heterogeneous structure and composition of bark as a platform to explore structure‐property relationships between its constituents. Likewise, the biorefining routes and, very often, analytical methods employed to analyze wood tissues are not applicable to bark. For example, confocal Raman spectroscopy has been extensively used to provide detailed maps of the chemical distribution of lignin and cellulose in wood at the cellular level, thus providing valuable information for wood biorefineries and materials [13, 14, 15, 16, 17]. Plant cell wall imaging by confocal Raman spectroscopy has been largely applied to lignocellulosic materials that do not contain large amounts of autofluorescent components, are light‐colored or have been extensively pretreated or bleached to remove those components. In this work, we advance the chemical mapping of challenging‐to‐resolve native bark tissues by employing stimulated Raman scattering (SRS) to overcome this barrier [18, 19, 20, 21].

Overcoming the autofluorescence barrier caused by the ubiquitous presence of polyphenolic compounds, coupled with rapid signal acquisition, allowed us to chemically map bark tissues at high resolution. Chemical interpretation is initially shown by the spectral profile of selected regions across the cellular structure, followed by classical least squares (CLS) and principal component analysis (PCA) to increase the sensitivity to complex overlapping data. Next, the removal of selected components, such as pectins, polyphenols, and lipophilic and hydrophilic extractives, further aids the spectral interpretation. The changes in chemical composition are linked to three‐dimensional structural impacts on morphological integrity shown by X‐ray microtomography and mechanical characterization, revealing how removal of different components affects the cohesion of the bark tissue. This perspective helps us understand the heterogeneity of bark to properly establish bark biorefineries. Compared to traditional biorefinery strategies, whereby, based simply on the material’s bulk chemical composition, one or another fraction is targeted and optimized for fractionation and valorization, our approach leads toward an understanding of the role of each component in the structural integrity of the material. By understanding the topochemistry and the physical impact of each of the components in pine bark across different scales, we propose the initial strategic steps toward structure‐first biorefineries.

## 2 Results and Discussion

### 2.1 Establishing the Topochemical Distribution of Bark Components

Pine bark (Figure 1a) differs greatly from wood both in chemical composition (Figure 1b) and in morphology (Figure 1c,d) [12, 22, 23, 24, 25, 26, 27]. Pine bark is formed by millimeter‐scale layered patches rich in fiber‐cellular structures. Between each layer, there are dense structures comprising mostly parenchyma cells oriented orthogonally to the growth direction. Such layered structures render pine bark with a flaky macroscopic appearance (Figure 1a). Within each layer, a well‐defined cellular ultrastructure is observed. The pine wood ultrastructure is rather simple, with tracheid cells dominating the wood tissue (Figure 1d). Pulping and other wood biorefineries have greatly benefited from a deep understanding of the wood anatomy and the chemistry of the wood cell wall [28, 29, 30, 31, 32, 33, 34].

**FIGURE 1:**
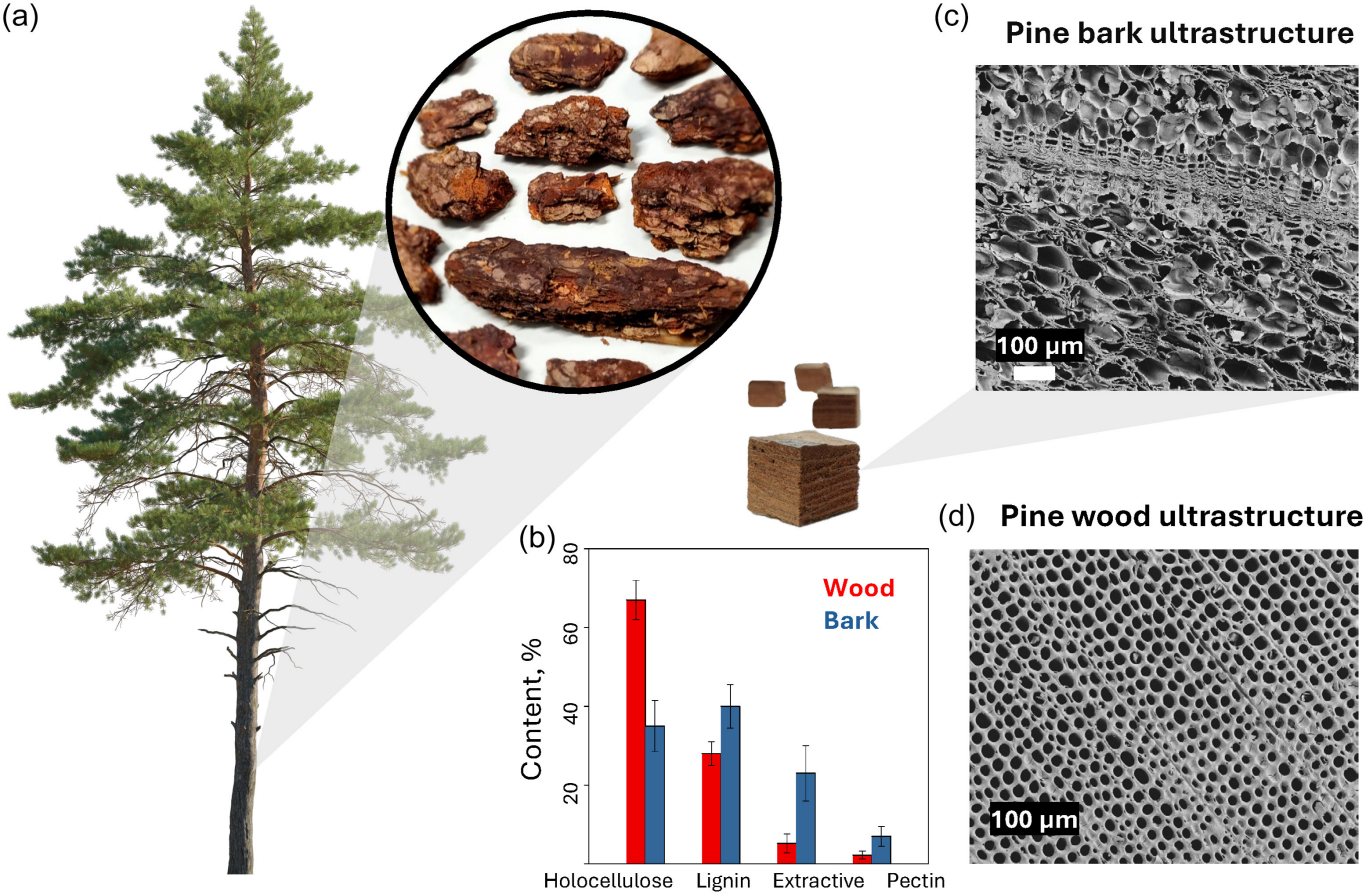
Analysis of the macrostructure, microstructure, and bulk chemical composition of pine bark, and comparison with pine wood. (a) Pine bark used in this study and its (b) chemical composition obtained at bulk, with a comparison to wood composition. (c) SEM of pine bark ultrastructure. (d) Comparative SEM of wood ultrastructure.

Pine bark has been analyzed extensively with wet chemistry methods [10, 11, 35] in bulk. In agreement with previous studies, pine bark used in this work had a content of ~40% lignin, ~30% holocellulose, 20%–25% extractives, and ~5% pectin (Figure 1b). Nevertheless, the topochemical distribution and the multidimensional structure–property relationships in the bark have been overlooked.

SRS [18, 36] was employed to map the chemical distribution of various compounds within bark and their variation at the cellular level. The SRS signal scales linearly with the concentration of the probed vibration, and SRS spectra match spontaneous Raman spectra. SRS provides an identical spectral response to spontaneous Raman scattering, thus allowing for spectral assignment and comparison to already existing large datasets. In this work, SRS intensities are used as relative, vector‐normalized measures to compare anatomical regions across the bark cellular structure before and after component removal. Chemical interpretation of the spectra across the cellular structure is further validated through CLS and PCA, as the multivariate approach increases the sensitivity toward complex overlapping data. Furthermore, the removal of selected components, leading to spectral changes, aids the spectral interpretation. We first acquired spectra (Figure 2a) from four different locations that represent bark at a cellular level, namely the cell corner, middle lamella, cell wall, and outer cell wall (Figure 2c). The outer cell wall, an ambiguous term in bark tissue, discussed here, corresponds to the layer in the cellular structure close to the lumen, as shown in Figure 2c—the highlighted dark blue area (note: this tissue should not be confused with the macroscopic outer bark). The spectral interpretation is based on reference component spectra, Raman bands identified in earlier works [37, 38], and on spectral changes observed after selective removal of bark components such as hydrophilic and lipophilic extractives, phenolic compounds, pectins, and hemicelluloses (Table S1). While the cell corner and middle lamella in wood have been shown to be dominated by lignin signals at ~1600 and ~1660 cm^−1^ corresponding to the aromatic ring stretching and C═O stretching of coniferaldehyde units, respectively, in bark, we observe more overlapping bands and a heterogeneous spectral profile requiring a more careful interpretation (Figure 2a). Spectra from the cell walls show 1092 and 2900 cm^−1^ bands corresponding to the cellulose‐rich region. Pectin is indicated by the 850 cm^−1^ shoulder appearing in the cell corner and less in the middle lamella spectra. The thick outer cell walls (dark blue in Figure 2c), a unique feature of bark cellular structure, show a similar spectral profile in the C─H stretching region (signal at 2935–2940 cm^−1^) to the cell corner and middle lamella. While in the 1600–1700 cm^−1^ range, outer cell walls show similarities to the cell wall. While this region possesses interesting features showing similarities to both the cell wall and the cell corner with the middle lamella, its complex overlapping spectra profile requires a more sensitive computational approach. Therefore, to better understand the composition of the outer cell wall, false‐color images were produced by using CLS (Figure 2d–f). CLS decomposes each pixelated Raman spectrum into contributions from selected reference spectra, in this case, spectra collected from cell corners and cell walls, producing a color map that shows the distribution across the whole image. The CLS image based on the cell corner spectra in Figure 2e shows compositional similarity between the cell corner, middle lamella, and outer cell walls. This evidence shows how cell walls are tightly interlocked between two highly polyphenol‐rich layers (Figure 2f). Such cellular interlocking is not seen in wood cells of the same species and suggests the origin of the recalcitrance and poor processibility of bark fibers. We then use PCA to gain more insight into spectral details. PCA increases the sensitivity of the analysis and reduces the noisiness as it mathematically summarizes the data from a large number of wavelengths to a few variables, principal components (Figure 2b). The first four scores (PC1–PC4) are overlapped in Figure 2g and shown in detail in Figure 2h–k. PC1 through PC4 account for 95% of variance explained. PC1 accounted for 61% of the total variation and is dominated by a strong positive band at 2900 cm^−1^, with another positive band at 1096 cm^−1^, highlighting the cellulose‐rich region. PC1 can be considered a representation of CH/CH2‐stretch‐producing carbohydrates. PC2 accounted for 28% of the total variation and showed broad positive signal peaking at 2940 cm^−1^ (asymmetric CH stretch of lignin methoxy groups), 1624 and 1656 cm^−1^ (C═C stretching of lignin coniferaldehyde units, chalcone‐like phenolics, and lipophilic extractives), with a 1660–1680 cm^−1^ shoulder (conjugated C═O stretching of α,β‐unsaturated carbonyl). This spectral profile highlights the cell corner, middle lamella, and the outer cell wall, dominated by conjugated C═C and C═O bonded structures of tightly cross‐linked lignin, chalcone‐type phenolics, and fatty and resin acids, holding the structure together. PC3 (4% of variation) showed strong positive bands at 1600 and 2945 cm^−1^ corresponding to aromatic ring stretch and aliphatic CH_3_ stretch of lignin, respectively. In comparison to PC2, PC3 seems to separate solely lignin from carbohydrates and extractives. PC4 (2% variance) is dominated by a broad positive 1684 cm^−1^ peak, highlighting the conjugated C═O stretching of α,β‐unsaturated carbonyl structures, separated from the negative signals at 1600 and 2945 cm^−1^ of cell wall polymers. These can be contributions from pine bark extractives such as resin acids and chalcone‐type phenolics, with a possible contribution from lignin coniferaldehyde carbonyls. PC5 and onward explained only 1% or less of the total variance and provided little useful chemical representation.

**FIGURE 2:**
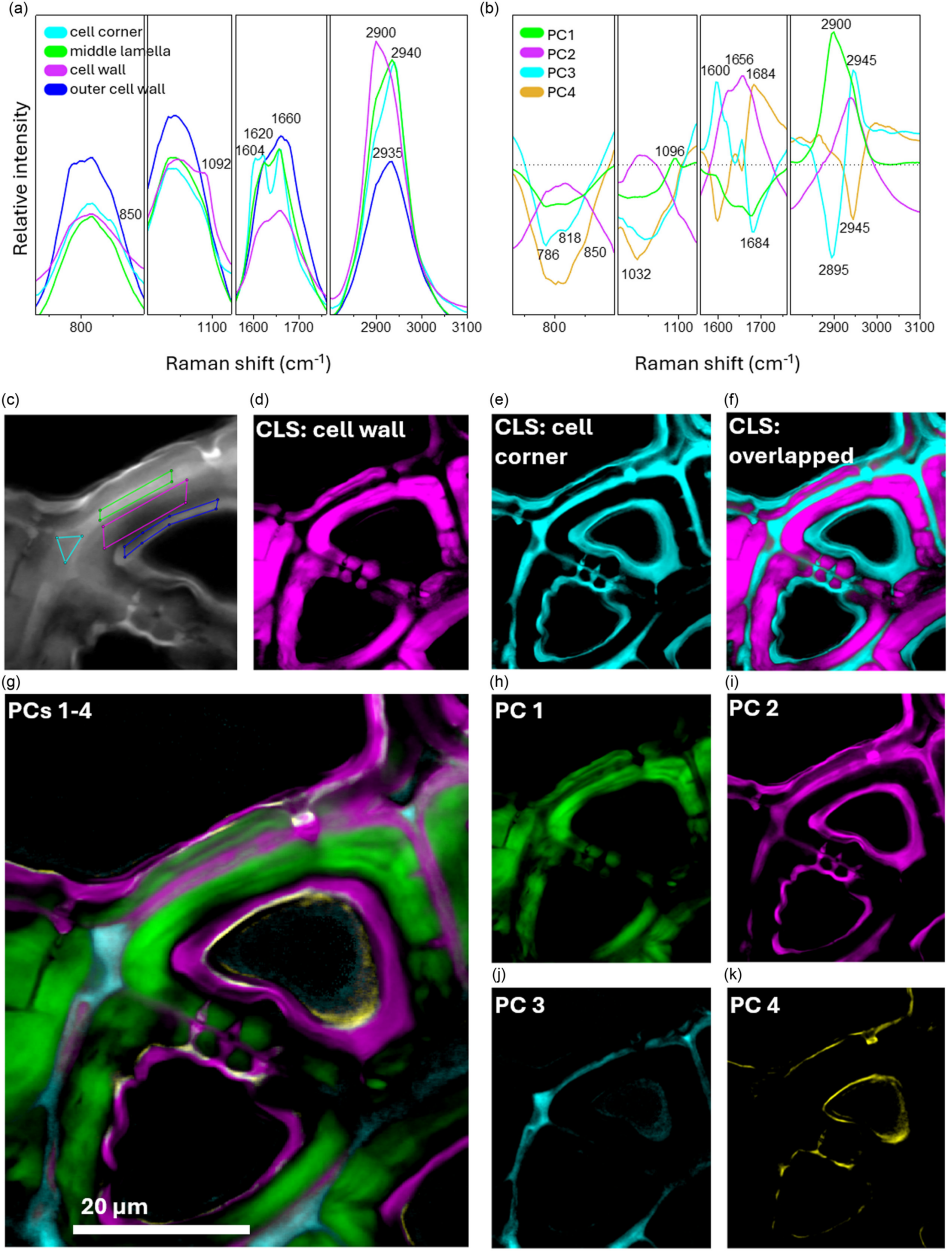
(a) Raman spectral profiles of the cell corner, middle lamella, cell wall, and outer cell wall. (b) principal component analysis (PCA) loadings. (c) Anatomical areas of choice. (d) Classical least squares (CLS)—integration based on average spectra of cell walls, (e) cell corners, and (f) their overlap. (g) Overlapped scores of the PCs 1–4, and scores of (h) PC1 (green), (i) PC2 (magenta), ( j ) PC3 (cyan), and (k) PC4 (yellow).

### 2.2 Interplay Between Various Selective Extractions and Topochemical Distribution

Efficiently loosening the ultrastructure of a given lignocellulosic precursor is central to high‐yield biorefineries. Therefore, we systematically and selectively removed bark components to monitor the associated changes in their topochemical distribution. Additionally, this data complements the spectral library and interpretation. Components have been extracted using various organic solvents (hexane, acetone, and ethanol), acidic and alkali conditions, water in reflux, or Soxhlet (to control the acidity of the medium). The mass loss acquired for residual bark blocks ranged from 5% to 50%, depending mostly on the extraction medium employed (Figure S1). We next focus on two extractions: i) all extractives removed (sequence of hexane and water) and ii) alkali‐treated material with significant mass loss and visual changes in the material.

For extractive‐free bark, major differences are seen in the 1600–1660 cm^−1^ region (Figure 3a). The extractives are largely removed from the cell wall and the middle lamella. As seen from CLS images based on average spectra collected from the cell corner and cell walls (Figure 3c–e), the outer cell walls remain unaffected and can still function as a cohesive structure. PCA (Figure S2) supported this as well: PC3 showed clear separation of the cell corner from cell walls and middle lamella on extractive‐free bark. In addition, selective separate removal of hydrophilic or lipophilic extractives shows that hydrophilic components are removed largely from the middle lamella and the cell wall, while lipophilic components are located across the bark cellular structure (Figure S3).

**FIGURE 3:**
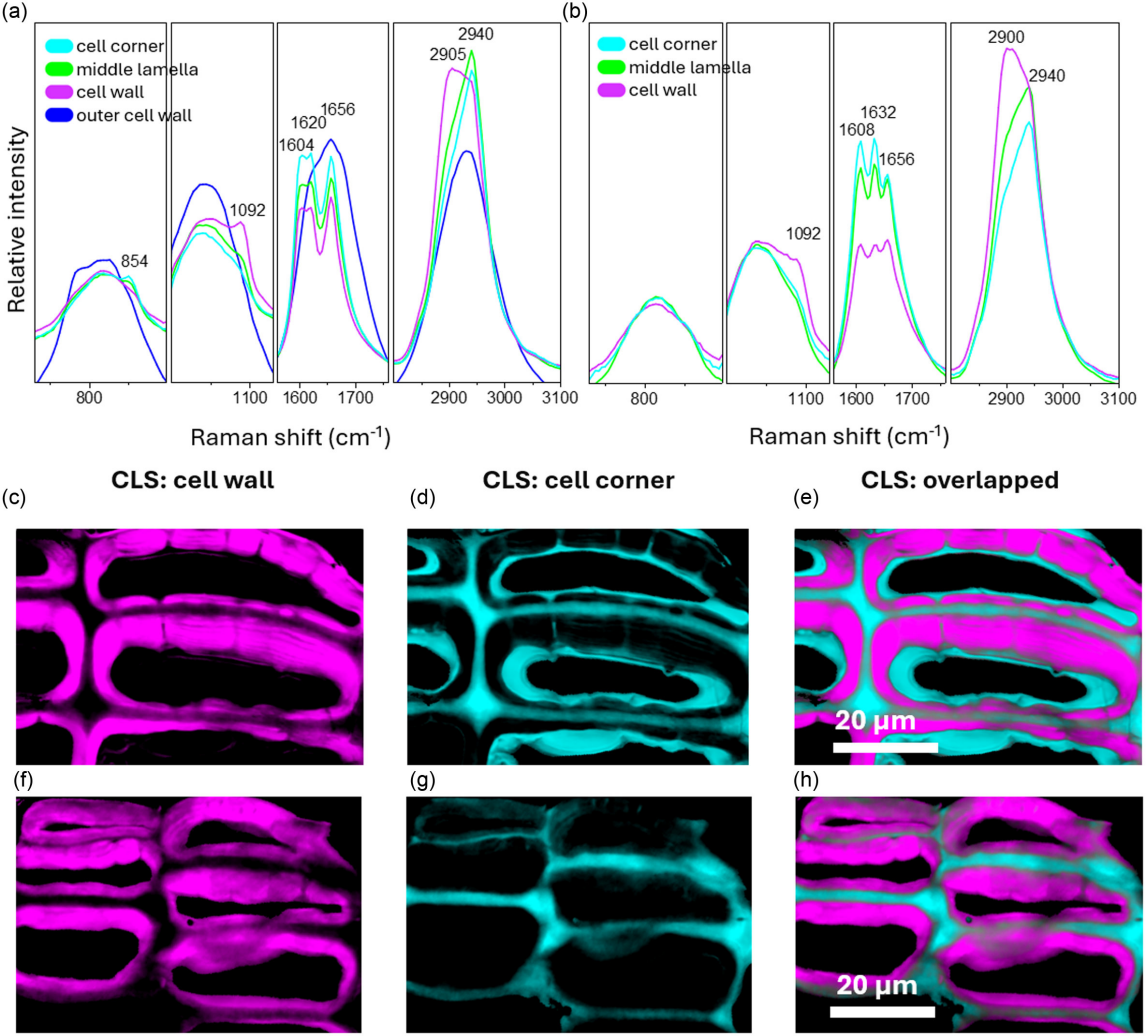
Raman spectral profiles of (a) extractive‐free bark and (b) alkaline‐treated bark. CLS—integration based on average spectra of (c) cell walls, (d) cell corners, and (e) overlapped for extractive‐free bark. Similarly, (f,g,h) for alkaline‐treated bark.

When more severe conditions are applied, such as in high‐alkaline or acidic treatments, the structural components are further removed. It should be noted that the increase in severity also leads to a decrease in the selectivity of the processes, as it is expected in the harsher alkaline and acidic treatments. Mild sulfuric acid treatment used for the removal of hemicelluloses also co‐extracts hydrophilic components. In addition, acidic conditions can lead to lignin fragmentation, repolymerization, and condensation [39, 40, 41]. In alkaline conditions, delignification is driven by the cleavage of ester linkages between lignin and the carbohydrates. These effects generally make cellulose fibers more accessible. While the main target of the alkaline extraction is the removal of polyphenolic compounds, extractives, both hydrophilic and lipophilic, and hemicelluloses are affected as well. Although extensive literature on those processes in bulk is available, in this study, we shed light into the cellular scale effects. Figure 3f–h show the changes in bark cellular profile caused by the disruption of the outer cell walls, which are no longer detected. The spectra of the cell corner, middle lamella, and cell wall resembled those of extractive‐free bark, except that the pectin signal at ~854 cm^−1^ was absent (Figure 3b).

In acidic environments, hemicelluloses are depolymerized by the hydrolysis of glycosidic bonds [42, 43]. Although polyphenols are not targeted for removal, their chemistry can undergo changes due to the condensation reactions leading to potentially undesirable and more stable forms in biorefineries [44]; thus, while performing this common biomass pretreatment strategy, one should consider possible side effects across different scales. FTIR spectra (Figure 4a) show a decrease of the signal at ~1740 cm^−1^ as the hemicelluloses and pectin are cleaved. Hemicelluloses are depolymerized and removed mostly from the cell wall (Figure 4c), making the cellulose fibers potentially more accessible. Pectin is removed selectively from the cell corner and middle lamella, observed by an 850 cm^−1^ signal decrease (Figure 4b). CLS based on the cell wall spectra of pectin‐free bark (Figure 4d) shows higher intensity, while CLS based on the cell corner spectra (Figure 4e) shows lower outer cell wall intensity than in sulfuric acid‐treated bark. This implicates pectin’s localized removal, which can potentially disrupt the double‐interlocking dynamics in bark.

**FIGURE 4:**
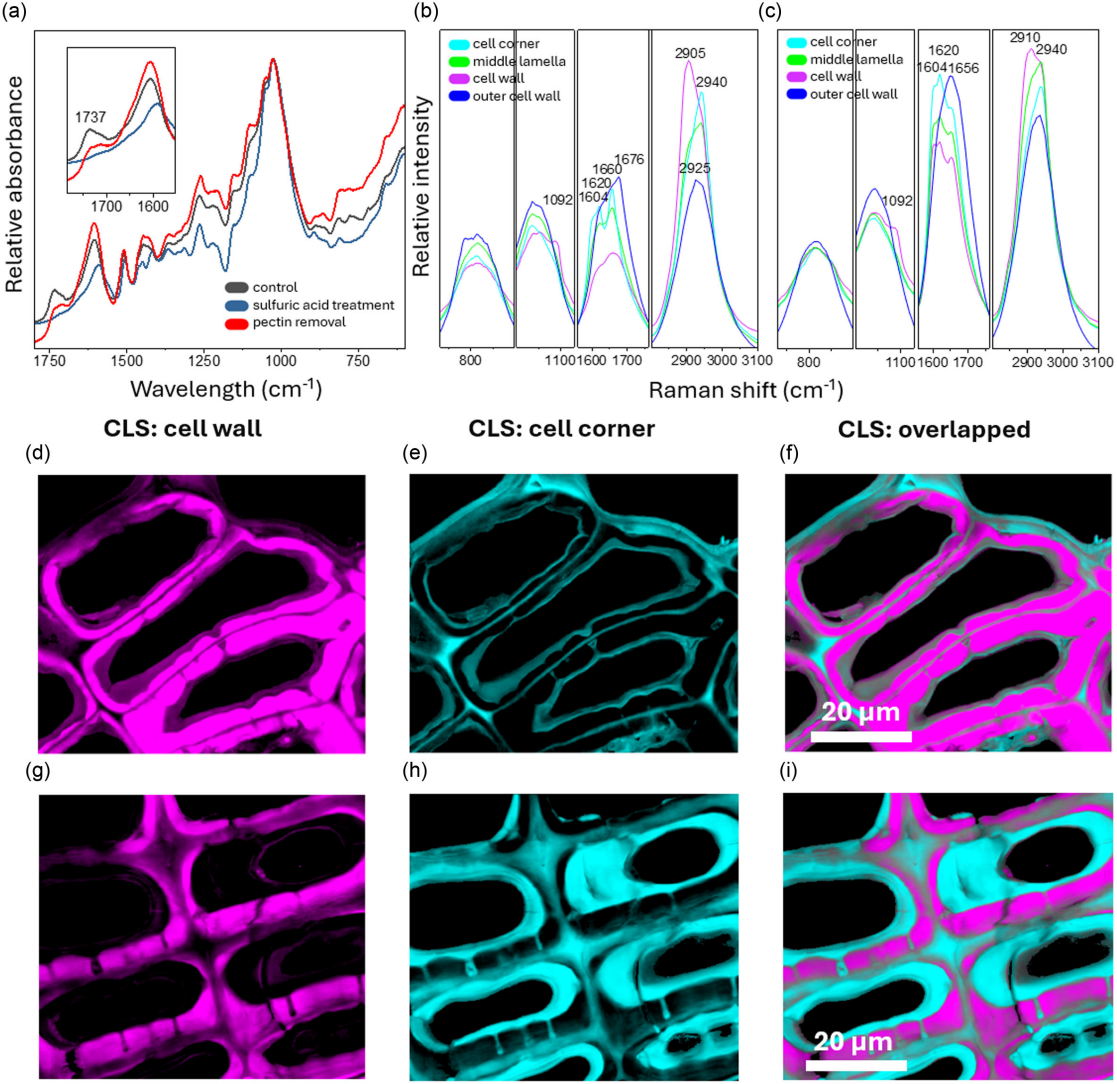
(a) FTIR and SRS spectra from different regions from (b) pectin‐removed and (c) sulfuric acid‐treated bark. CLS images from the samples with (d–f) pectin removed, (g–i) sulfuric acid treatment.

### 2.3 Effect of the Removal of Bark Components on Its Structural Cohesion and Morphology

Differences observed at the microstructural level are then correlated with macrostructural cohesion by employing compression tests on native and extracted bark blocks with *ca*. 1 cm^3^. While smaller particle sizes, such as ground bark, inherently increase extraction yields compared to larger intact pieces, the mechanical compression and X‐ray microtomography analyses on solid blocks provide specific insights into fiber–fiber and interfacial interactions within the native tissue. In addition, using larger pieces is more representative of industrial practices as fine grinding requires significant energy input. Nevertheless, direct, one‐to‐one extrapolations across these different sample formats (milled particles, solid blocks, and microsections) should be carried out with caution.

The removal of all extractives decreased the compression strength by 10% (Figure 5a). Interestingly, removal of only lipophilic and only hydrophilic extractives gave an increase of 9% and a decrease of 25%, respectively. An increase in compression strength after the removal of lipophilic components might be due to the better cohesion of cellulose fibers with each other and a more intact structure. While the hydrophilic components are removed primarily from the middle lamella area, as seen from SRS spectra (Figure S3), they loosen the middle lamella and lead to a decrease in strength. Removal of hydrophilic extractives was done under reflux, where extracted phenolic acids lower the pH of the medium down to 3–4, and in addition, with Soxhlet extraction, where the neutrality of the medium is kept. In more acidic conditions, the compression strength decreased by 6% compared to 25% in neutral conditions. This might be due to the condensation of lignin and the formation of new C─C bonds. Also at the macroscale, the effects on the porous structure of bark caused by different treatments were investigated (Figure 6). Complete removal of extractives increased both the porosity and mean pore size by 7% and 16%, respectively (Figure 6a,e). We observed how the removal of lipophilic components led not only to a higher porosity of the bark block but also to a greater mechanical strength. While seemingly a contradictory trend, as discussed above, this can be caused by increased cohesion between fibers upon the removal of lipophilic structures. Removing the hydrophilic components gave a decrease in mechanical strength while maintaining the same porous organization.

**FIGURE 5:**
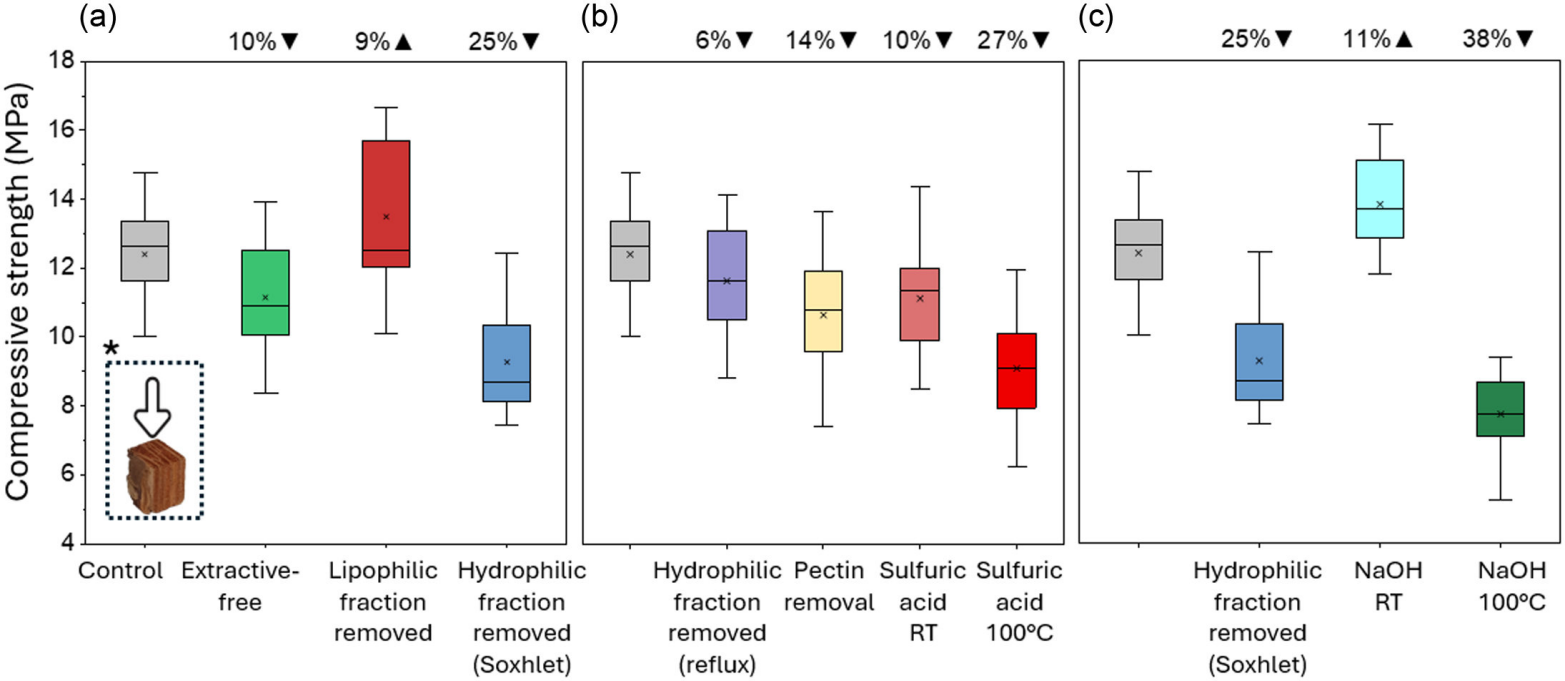
Uniaxial compression strength of bark blocks after (a) different extractive fractions removed: all extractives removed (successive hexane and water Soxhlet extractions); lipophilic—hexane soluble extractives removed; hydrophilic—water‐soluble fraction removed under neutral pH via Soxhlet extraction. (b) neutral to acidic aqueous treatments: hydrophilic—water‐soluble fraction removed under reflux conditions where the pH drops down to 3–4; pectin removal via citric acid under pH 2; 1% sulfuric acid treatment at RT; 1% sulfuric acid treatment at 100 °C. (c) neutral to alkaline aqueous treatments: hydrophilic—water‐soluble fraction removed under neutral pH via Soxhlet extraction; 1% NaOH at RT; 1% NaOH at 100 °C. *An example of a bark block used, the direction of the compression test; the sample size *n* = 20–30. The values on the top of the figure represent relative to the control sample (gray boxes) changes in compression strength.

**FIGURE 6:**
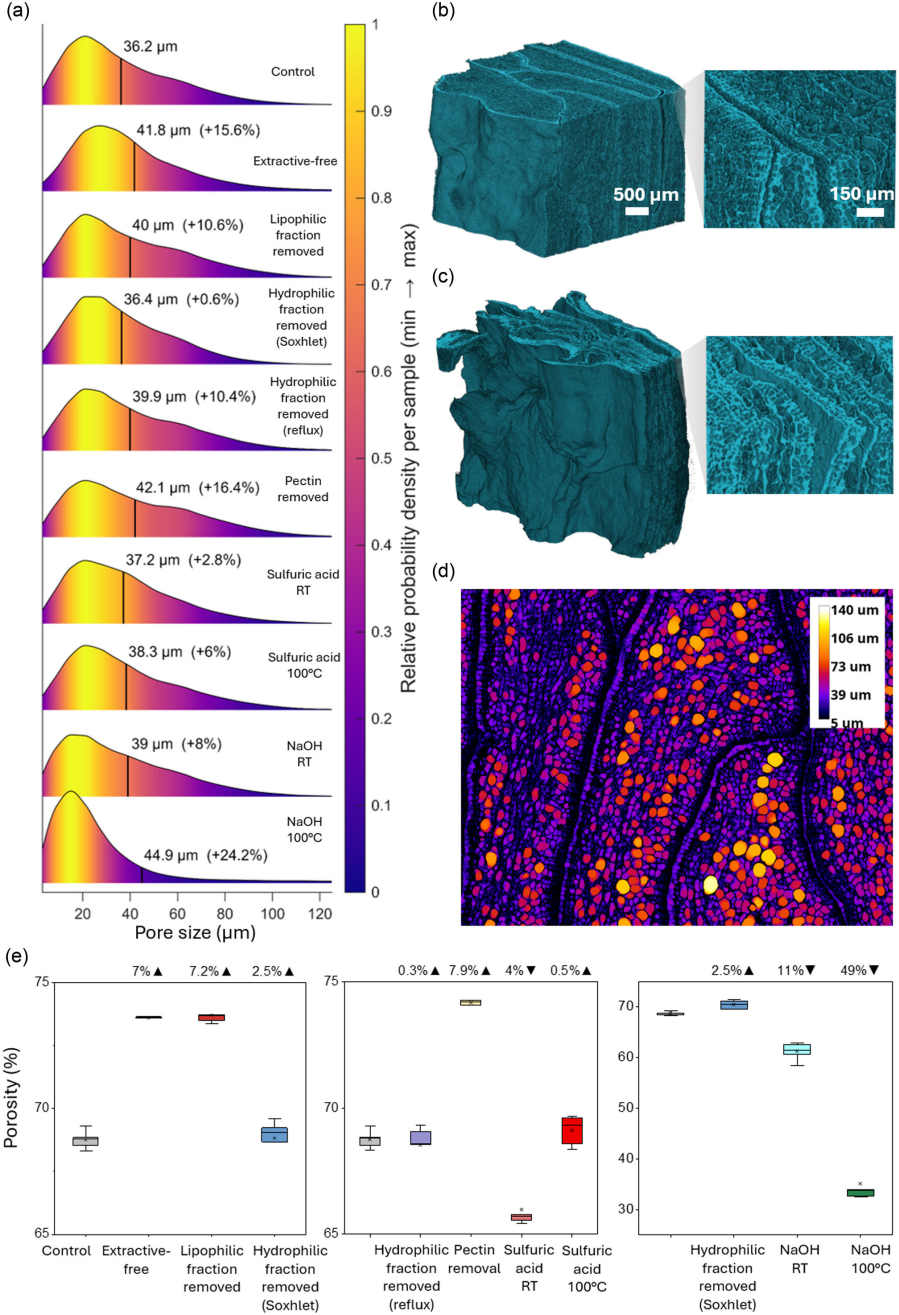
(a) Pore size distributions of bark blocks after different fractions removed: all extractives removed (successive hexane and water Soxhlet extractions); lipophilic—hexane‐soluble extractives removed; hydrophilic—water‐soluble fraction removed under neutral pH via Soxhlet extraction; hydrophilic—water‐soluble fraction removed under reflux conditions where the pH drops down to 3–4; pectin removal via citric acid under pH 2; 1% sulfuric acid treatment at RT; 1% sulfuric acid treatment at 100 °C; 1% NaOH at RT; 1% NaOH at 100 °C; the mean pore sizes and relative to control bark changes (%) are shown over each histogram. (b) X‐ray microtomography 3D visualization and porous structure of a bark block and (c) after 1% NaOH 100 °C treatment. (d) Bark cross‐section showing pore size distribution. (e) Porosity of bark blocks after different fractions are removed: different extractive fractions are removed; neutral to acidic aqueous treatments, and neutral to alkaline aqueous treatments. The % changes at the top axis are relative to the control sample.

Removal of pectin with citric acid (at pH 2) led to a decrease of 14% in the compression strength (Figure 5b). Additionally, it had a similar impact on the porosity and pore sizes of bark as did the removal of all extractives (Figure 6a,e). Even with just ~5% of mass lost with pectin removal compared to the ~25% of extractives, the physical impacts are more significant, highlighting the structural importance of pectin. Further stronger acidic treatments led to depolymerization and dissolution of hemicelluloses, affecting structural integrity more and leading to a 27% decrease in the compression strength with up to 28% mass loss. Similarly, the compression strength of bark blocks decreased with alkaline treatment, as expected, due to the removal of up to 50% of the mass that includes structurally vital components. On a general level, bark tissues are largely dominated by pores in the range of 15–40 micrometers (Figure 6a,d). At the same time, the shape of the distribution changes radically, with small pores starting to dominate (Figures S7 and S8). This indicates that most of the changes occur at the cellular level, not causing extensive damage to the macrostructure of the bark. Only more severe conditions, with NaOH treatment, under 100 °C, have led to a remarkable change in the porous organization. This is shown as a radical change in the shape of the pore size distribution, 24% mean pore size increase, and 49% porosity decrease due to the densification of the bark tissue after the removal of polyphenolic compounds (Figure 6a,c). However, with mild alkaline treatment at RT, where a smaller fraction of polyphenols is removed, the compression strength rises (Figure 5c). The increase in compression strength might be due to the removal of components just in amounts without disrupting their structure while leading to better cohesion between the fibers. In addition, slight increases in compression strength can be observed with nonselective extractive removal (Figure S5). Thus, while pre‐removing bark components in biorefineries, it should be considered that not just the amounts but their nature and composition are of importance.

The structure‐first concept is proposed as a framework for filling the gaps in the fundamental understanding of this chemically heterogeneous material at different scales. While SRS and X‐ray microtomography serve to establish how components control tissue cohesion, they are not applied here and intended for monitoring industrial processes but to aid the design of novel industrial strategies. Pine bark, as a heterogeneous lignocellulosic material, is used here as a platform to monitor and identify different trends across scales. Future work could be the application and validation of this strategy on other industrially relevant biomasses of topochemistries as complex as those observed in pine bark. Structure‐driven design of biorefineries can be implemented to avoid and anticipate unexpected and undesirable side effects leading to increased biomass recalcitrance and at the same time benefit from the inherent functionality of the native components.

## 3 Conclusion

Through the topochemical analysis of bark tissues and macroscale texture and integrity impact responses, we provided a multiscale platform to fill the gaps in the understanding of this multifunctional natural material. The SRS imaging showed how the bark fibers are double‐interlocked at the cellular level, leading to the known poor processability. The outer cell walls are highlighted by conjugated C═C double bonds and unsaturated carbonyl structures that shield the bark fibers. The distribution of extractives shows distinct roles for hydrophilic and lipophilic components. Pectins distributed throughout the cell corner and middle lamella could be targeted to loosen the bark structure, followed by the removal of selected extractives. Although not just the target components but the solvent selection and pH of the medium play a significant role. The SRS spectra showed the vital role of outer cell walls and the possibility to attack those bonds in order to liberate the fibers. The cellular‐level changes were correlated to macroscopic structural impacts, showing how the nature and not just the amounts of constituents removed differently affect the integrity and cohesion in bark fibers. Thus, the hierarchical organization of components of different nature in bark can lead to the design of materials in a similar pattern and a tailored combination of selective treatments on bark for its efficient deconstruction. With this work, we contribute to filling the gap in the understanding of the structural role and contribution of bark components through a topochemical analysis in order to push forward the full utilization of such bioresources. We offer the valorization of this major forestry by‐product based on its inherent functionality, designing structure‐first biorefineries.

## 4 Materials and Methods

### 4.1 Materials

Bark was separated from a 70‐year‐old Scots pine at 1.5 m height in Southern Finland and stored frozen. Fine bark with a 1 mm particle size was produced by grinding in a Retsch SM 300 Cutting Mill and freeze‐dried. Bark blocks of 1 cm a side were cut with a circular saw and stored at 21 °C and 65% humidity in a climate‐controlled room until a constant weight. Moisture content was determined by drying in 105 °C. Cross‐sections of 20 μm thickness were prepared with a rotary microtome (RM 2255, Leica) and put on glass microscope slides; a few drops of deionized water were added, and glass cover slips were placed on top.

### 4.2 Bark Extractions

The ground bark, bark cross‐sections, and bark blocks were treated to remove different compounds following treatments. Bark was refluxed in excess amounts of solvents (hexane and water) with a 200 to 1 liquid‐to‐solid ratio to obtain extractive‐free bark. Soxhlet extractions were done with the same liquid‐to‐solid ratio. Solvent exchange to water was carried out on bark blocks before storage. Alkaline treatment with a sodium hydroxide concentration of 1% and sulfuric acid treatment at a 1% concentration were done at 20 °C room temperature and 100 °C under reflux. A citric acid solution at pH 2 was used for pectin removal at 80 °C.

### 4.3 Uniaxial Compression Strength

The mechanical properties of bark blocks were evaluated by uniaxial compression using a TA. An XTplusC Texture Analyzer equipped with a 500 g load cell. The measurements were carried out at a compression rate of 0.5 mm s^−^^1^, within a controlled environment (21 °C and 50% relative humidity) and along the longitudinal direction. The ultimate compression strength value from each stress–strain profile was determined and reported as an averaged value.

### 4.4 Stimulated Raman Hyperspectral Microscopy and Data Analysis

The SRS spectra were measured with an in‐house developed spectral focusing coherent Raman microscope [18]. In brief, the instrument consists of an Olympus FV3000 confocal laser scanning microscope coupled with a dual output tunable fs laser (InSight X3+, Spectra‐Physics) and spectral focusing unit (SF‐TRU, Newport). Hyperspectral SRS images were measured with a UPLSAPO 60×W1600 NA 1.20 objective over 4 spectral regions: 730–900, 1000–1130, 1550–1760, and 2800–3100 cm^−1^. The Stokes wavelength was 1045 nm, and the pump wavelength 960, 940, 888, and 798 nm for each of the spectral regions, respectively. The laser power levels at the sample were approximately 100 mW for the Stokes and 14 mW for the pump but varied slightly over the different regions, with lower wavenumber regions having higher laser power to compensate for the weaker Raman vibrations. The transmitted light was collected with a high numerical aperture condenser lens (NA 1.4, Leica), and the SRS signal was detected with an APE SRS lock‐in unit. The signal intensity on the first two regions is weaker; thus, for easier visualization, the intensity is multiplied by 4 for those in the figures. Data was pre‐processed by the BM4D algorithm [45] to reduce noise, with the background removed by thresholding the inner lumen areas, vector normalization (*p* = 1), and mean centering. Preprocessing and processing, including non‐negative CLS and PCA, were done in in‐house developed MATLAB‐based analysis software. Spectra were exported and plotted in OriginPro 2025.

### 4.5 Attenuated Total Reflectance‐Fourier Transform Infrared (ATR‐FTIR) Spectroscopy

ATR‐FTIR spectra were obtained using a Spectrum Two FT‐IR Spectrometer with a LiTaO_3_ detector (PerkinElmer, USA). The resolution was 4 cm^−1^, and 32 scans were accumulated over the range of 600–4000 cm^−1^ for each sample.

### 4.6 X‐Ray Microtomography

Tomographic imaging was performed using a custom‐built JTomo microtomography system equipped with a Hamamatsu L12161‐07 microfocus X‐ray source and a Teledyne Dalsa Shad‐o‐Box 6K HS detector (2940 × 2304 pixels) [46]. The X‐ray source was operated at 40 kV and 100 µA without filtration. A camera exposure time of 4.5 s per projection was used. In total, 2940 projections were acquired over a full 360° rotation using continuous acquisition. A geometrical magnification of 19.6 was applied, resulting in an effective pixel size of 2.53 µm. Image reconstruction was performed using a filtered back‐projection algorithm [47]. The reconstructed images with an isotropic voxel size of 2.5 µm were used to assess porosity, characterize pore size distributions, and visualize the internal structure of the samples in 3D. Image noise was reduced using a bilateral filter with a radiometric sigma of 2000 and a spatial sigma of 2 voxels. Segmentation of the 3D images was performed in Fiji using Otsu thresholding. A global threshold was applied to generate binary images, where the solid phase and pore space were represented by white and black voxels, respectively. Measurements were taken from a rectangular region covering most of each sample. Pore size characterization was conducted using local thickness analysis. In this method, the local thickness at each pore voxel is defined as the diameter of the largest sphere that fits entirely within the pore space and contains the voxel of interest [48]. This approach provides a volume‐based description of pore dimensions without requiring the identification or segmentation of individual pores. Pore size distributions were derived by classifying local thickness values into size intervals with a bin width of 6 µm. As this method is based on volumetric thickness measurements rather than the identification of discrete pores, the resulting distributions describe the volume fraction associated with each size class rather than the sizes of individual pore objects. In addition, for elongated pores, the pore size determined in this way corresponds typically to their transversal diameter. All image processing and quantitative analyses were performed using pi2 [49]. For visualization purposes, binary pore structures were scaled to a voxel size of 5 µm prior to three‐dimensional rendering conducted using MeVisLab.

### 4.7 Scanning Electron Microscopy

The morphology of the cross‐sectional areas of pine bark and wood was examined using scanning electron microscopy (SEM) (Zeiss Sigma VP). The samples were coated with a thin layer of gold/palladium alloy using a Leica EM high‐vacuum sputter coater. SEM images were acquired at an accelerating voltage of 5 kV.

### 4.8 Characterization of Extracts

Extractives were analyzed by GC‐MS following the reported protocol [50]. Briefly, 2 mg of dry sample was dissolved in 100 µL DMF and 10 µL pyridine with 50 µL BSTFA with 10% trimethylchlorosilane and heated at 60 °C for 15 min and shaken occasionally. Compounds were identified via GCMSsolution and LabSolutions software with the NIST 2017 (National Institute of Standards and Technology) GC‐MS mass spectral library.

## Author Contributions

**Rustam Ibadov** **:** conceptualization, methodology, investigation, formal analysis, data curation, visualization, writing – original draft, writing – review and editing. **Teemu Tomberg** **:** investigation, formal analysis, and data curation. **Muhammad Awais** **:** formal analysis, writing – review and editing. **Idil Ertem** **:** formal analysis and data curation. **Joni Parkkonen** **:** investigation. **Paavo Penttilä** **:** writing – review and editing. **Monika Österberg** **:** writing – review and editing, resources, funding acquisition, sand supervision. **Bruno D. Mattos** **:** conceptualization, methodology, funding acquisition, resources, supervision, writing – original draft, writing – review and editing.

## Funding

This study was supported by Opetus‐ ja Kulttuuriministeriö (VN/3137/2024‐OKM‐6).

## Conflicts of Interest

The authors declare no conflicts of interest.

## Supporting information

Supplementary Material

## Data Availability

All data are included in the Supporting Information. Any other data are available on request from the authors. Supporting Information is available.
