# Supplementary material for: Topochemical Analysis of Pine Bark toward Structure‐First Biorefineries
Source: ChemSusChem. 2026 Nov 12;19(22):e71220. doi: 10.1002/cssc.71220 (PMC13757301; doi:10.1002/cssc.71220)
Supplement: Supplementary file 1 [file CSSC-19-e71220-s001.pdf]

Supporting information

Topochemical Analysis of Pine Bark Towards Structure-First Biorefineries

Rustam Ibadov[1], Teemu Tomberg[2], Muhammad Awais[1], Idil Ertem[3], Joni Parkkonen[3], Paavo Penttilä[3], Monika Österberg[1*], Bruno D. Mattos[1*].

[1] Department of Bioproducts and Biosystems, School of Chemical Engineering, Aalto University, Vuorimiehentie 1, Espoo FI-00076.

[2] Division of Pharmaceutical Chemistry and Technology, Faculty of Pharmacy, University of Helsinki, Helsinki, Finland.

[3] Department of Physics, Nanoscience Center, University of Jyväskylä, Survontie 9, FI-40500 Jyväskylä, Finland.

** Corresponding authors: Bruno D. Mattos (bruno.mattos@aalto.fi), Monika Österberg (monika.osterberg@aalto.fi)*

This file contains 8 supporting figures, 2 supporting tables and 1 supporting discussion in 15 pages.

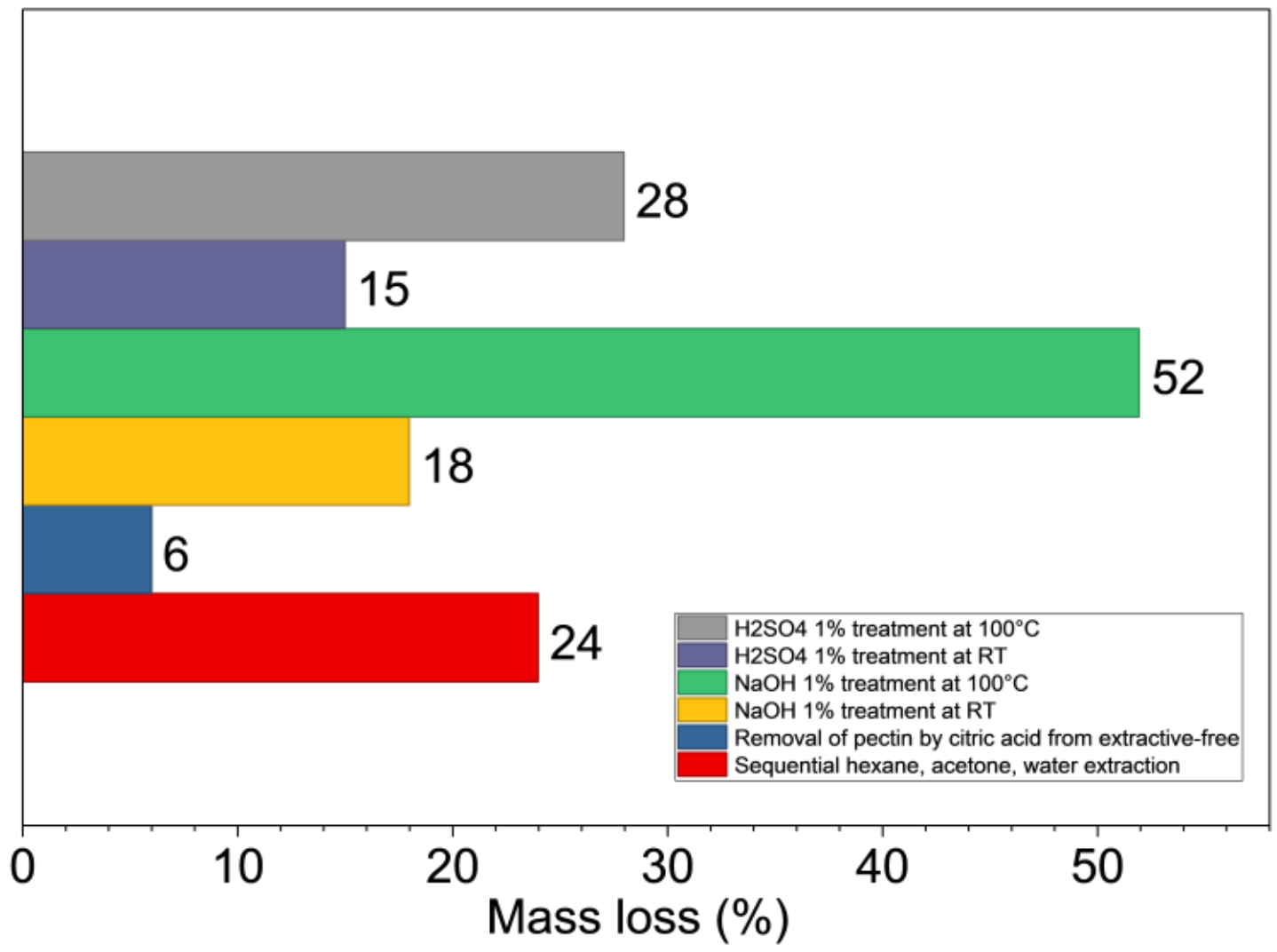

Figure S1. Mass losses during different treatments.

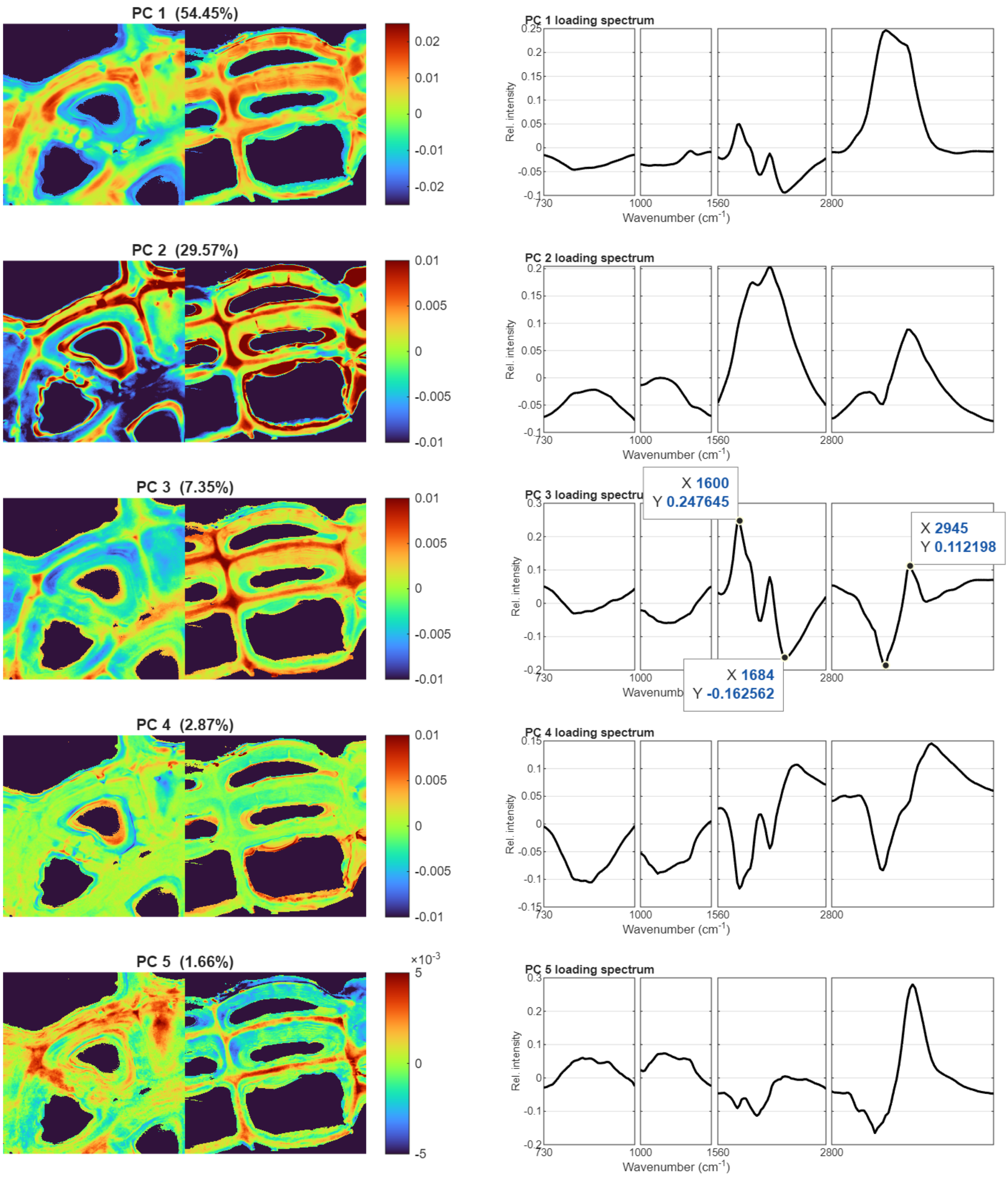

Figure S2. PCA on raw bark (left-hand color image) and extractive-free bark (right-hand color image); PC loadings. PC3 shows negative 1684 cm^{-1} signal, blue colored area, highlighting the cell walls, on the left image (control bark), which isn't observed on the extractive-free image (right). On the extractive-free image, 1600 cm^{-1} signal, red colored, shows cell corner and the middle lamella, on control bark, cell corner only.

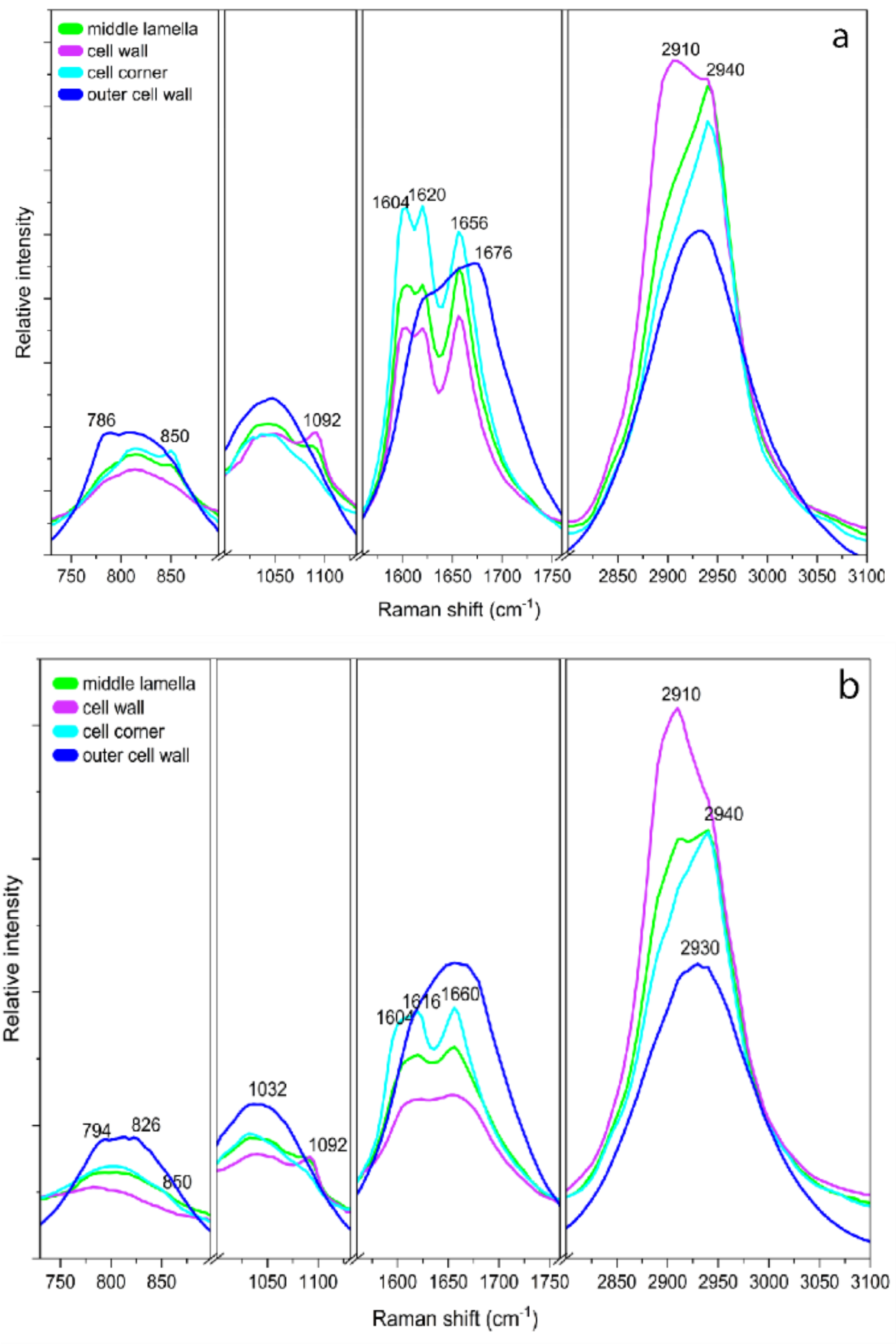

Figure S3. SRS spectra from different regions from lipophilic extractives removed (a) and hydrophilic extractives removed (b) bark.

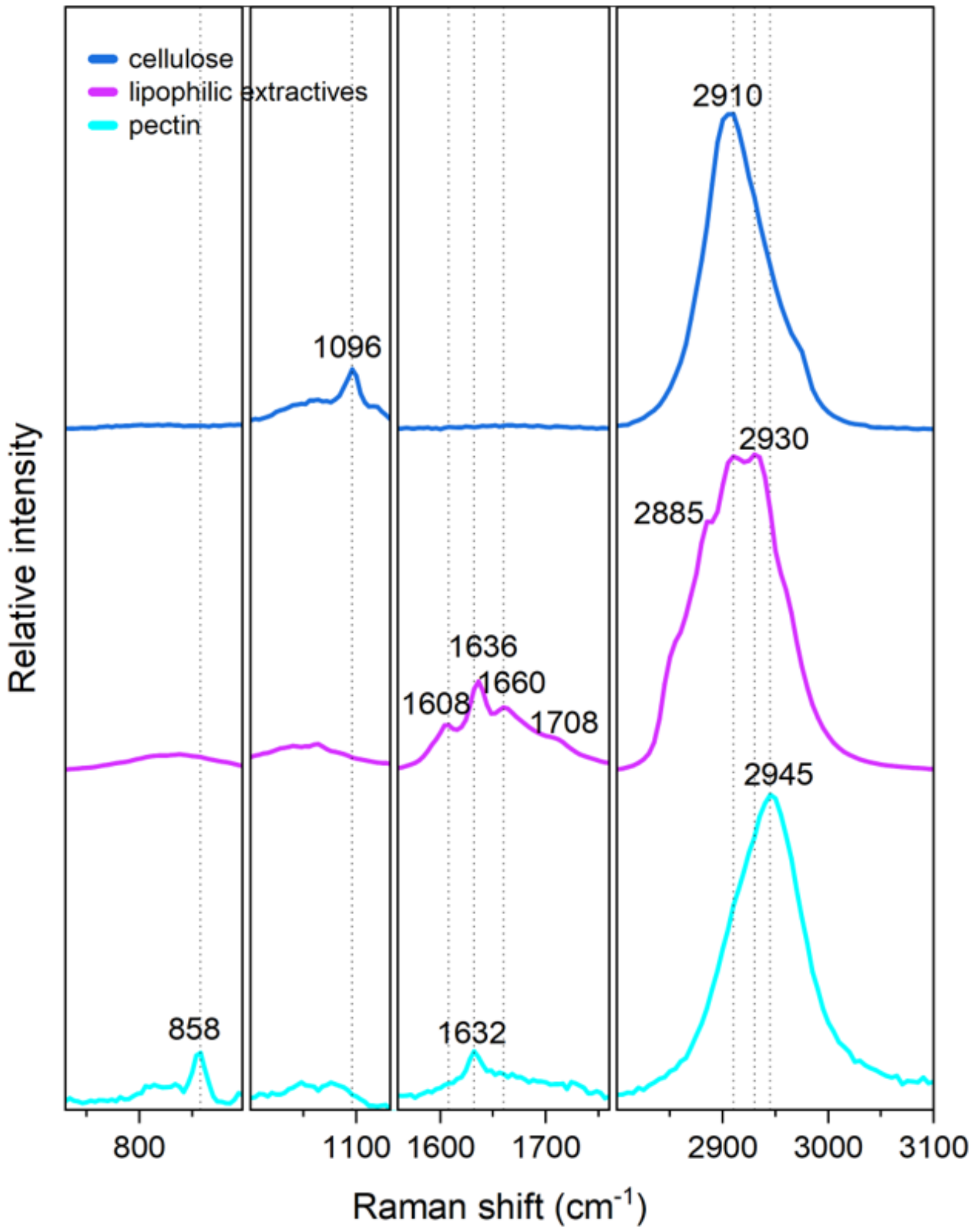

Figure S4. SRS spectra of reference compounds.

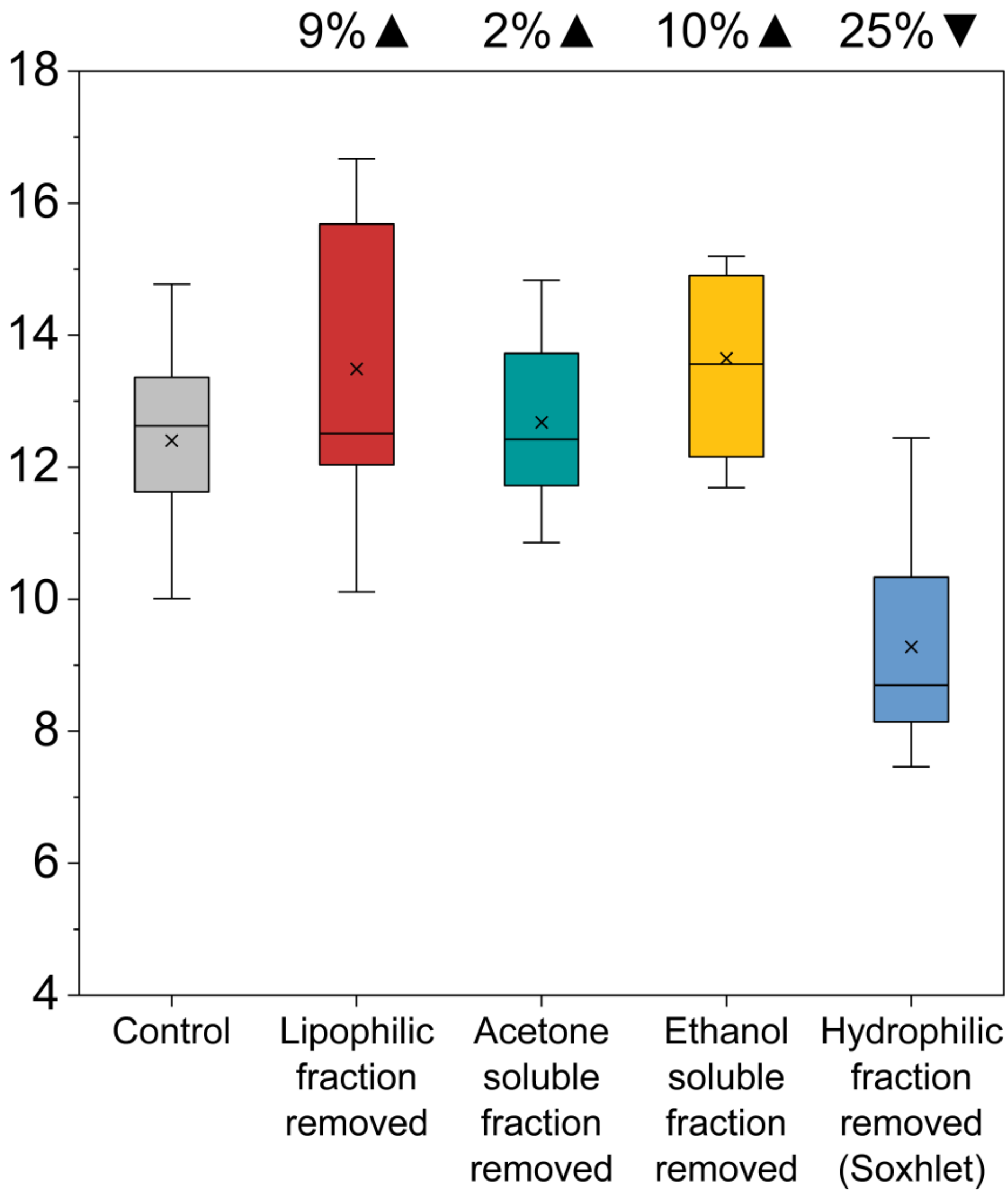

Figure S5. Compression strength (MPa) of bark blocks after hexane, acetone, ethanol, water treatments. Top axis shows relative to control sample changes (%).

a. GC-MS profile of hexane soluble extractives

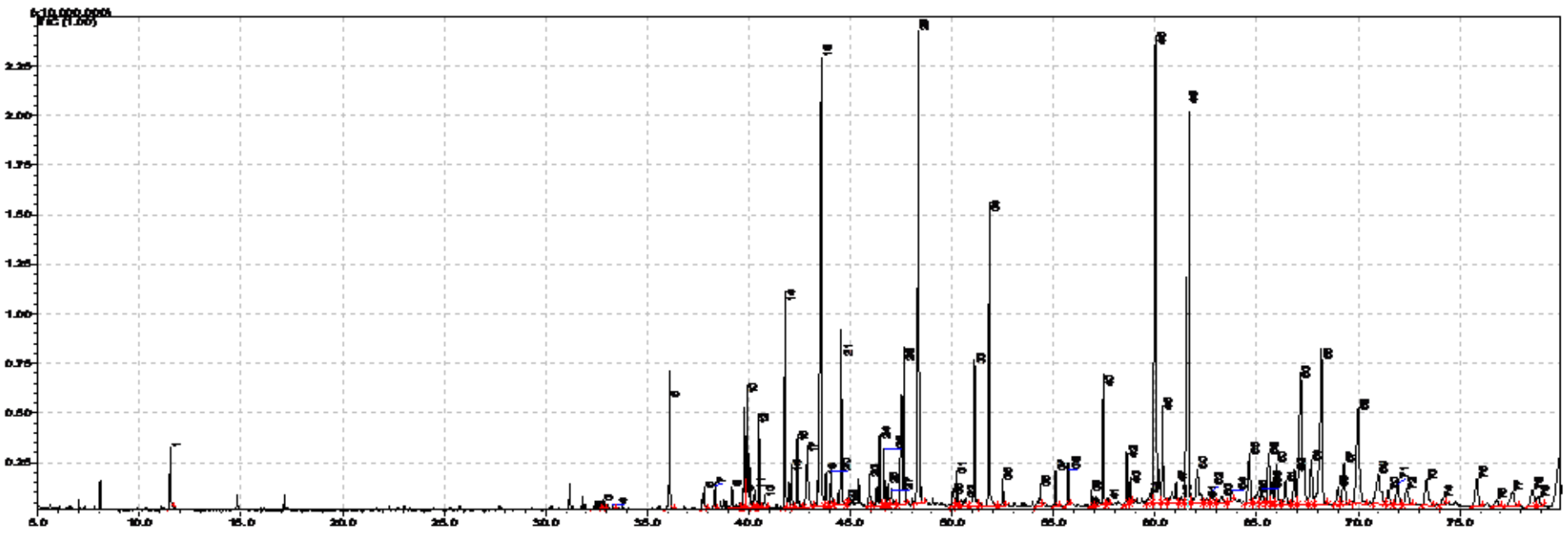

b. GC-MS profile of water-soluble extractives

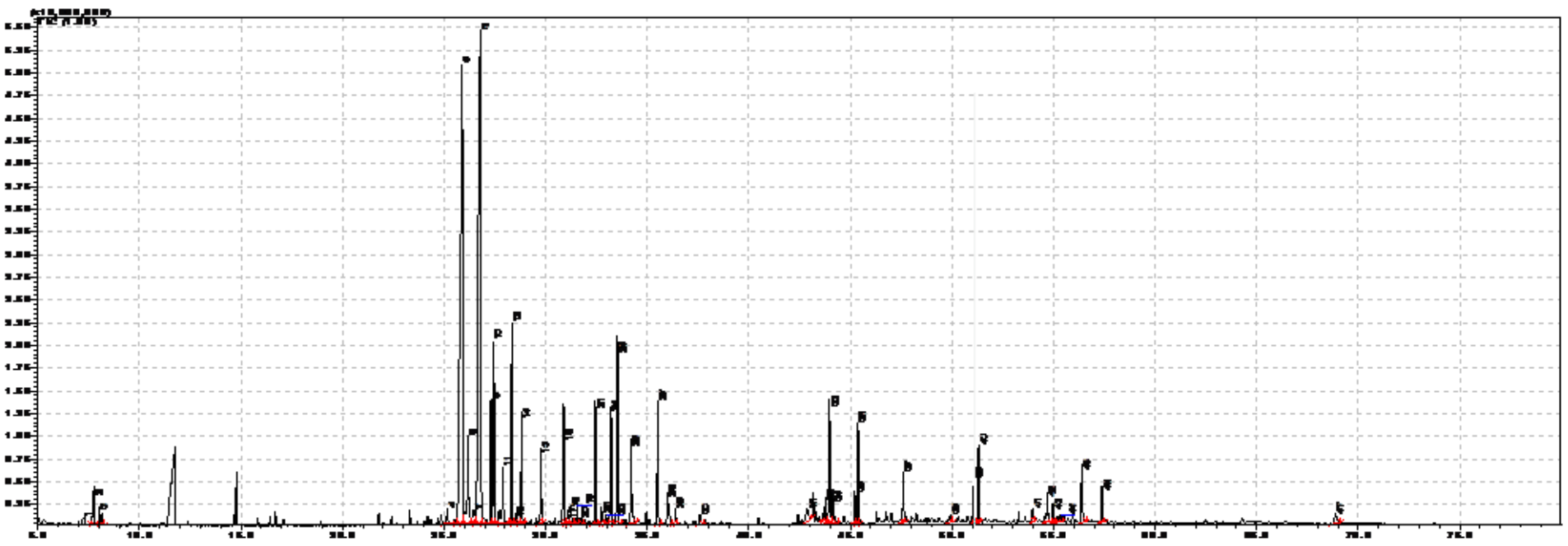

c. GC-MS profile of acetone soluble extractives

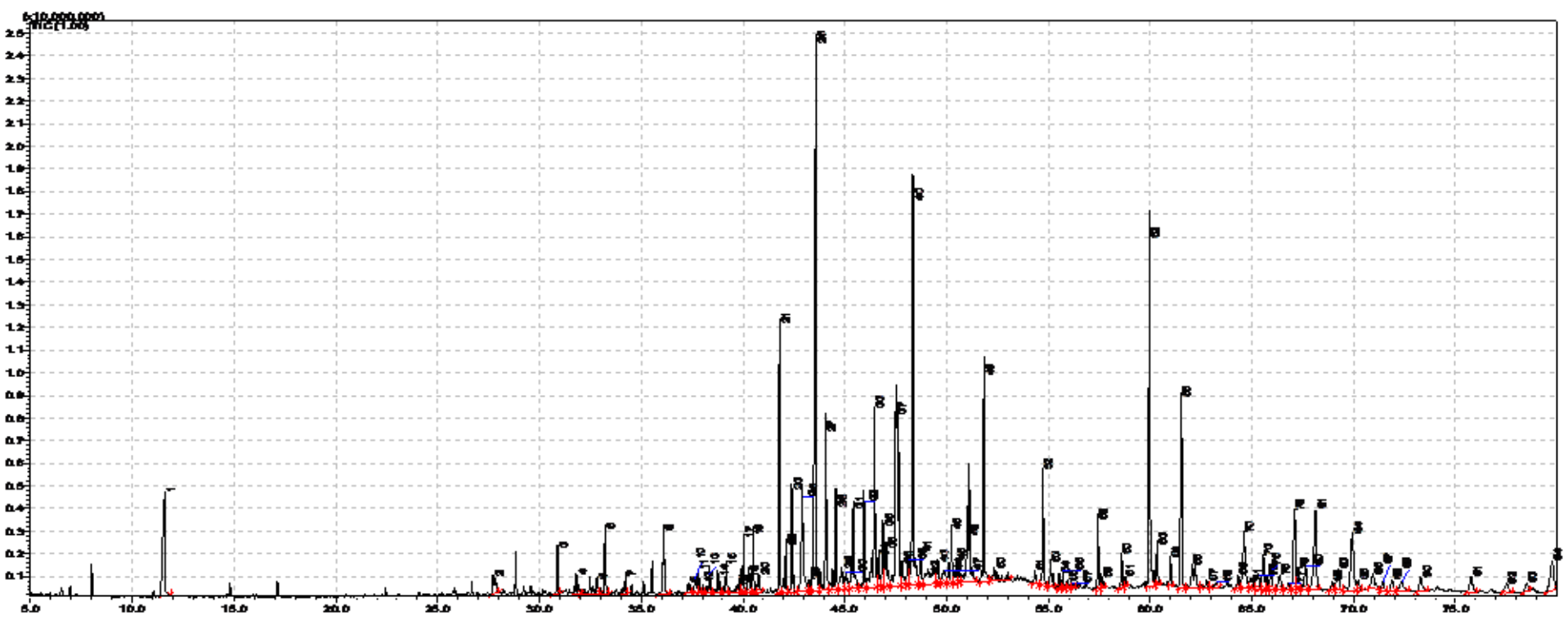

d. GC-MS profile of ethanol soluble extractives

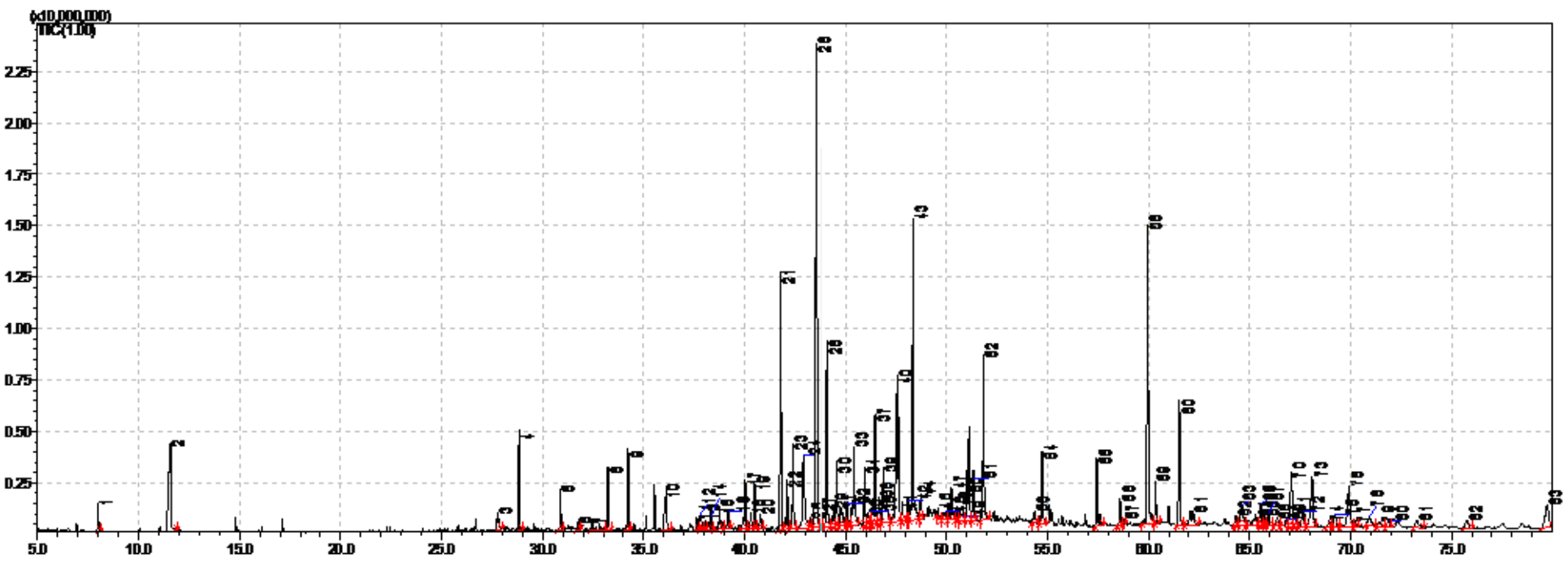

Figure S6. GC-MS spectral profiles of extractives.

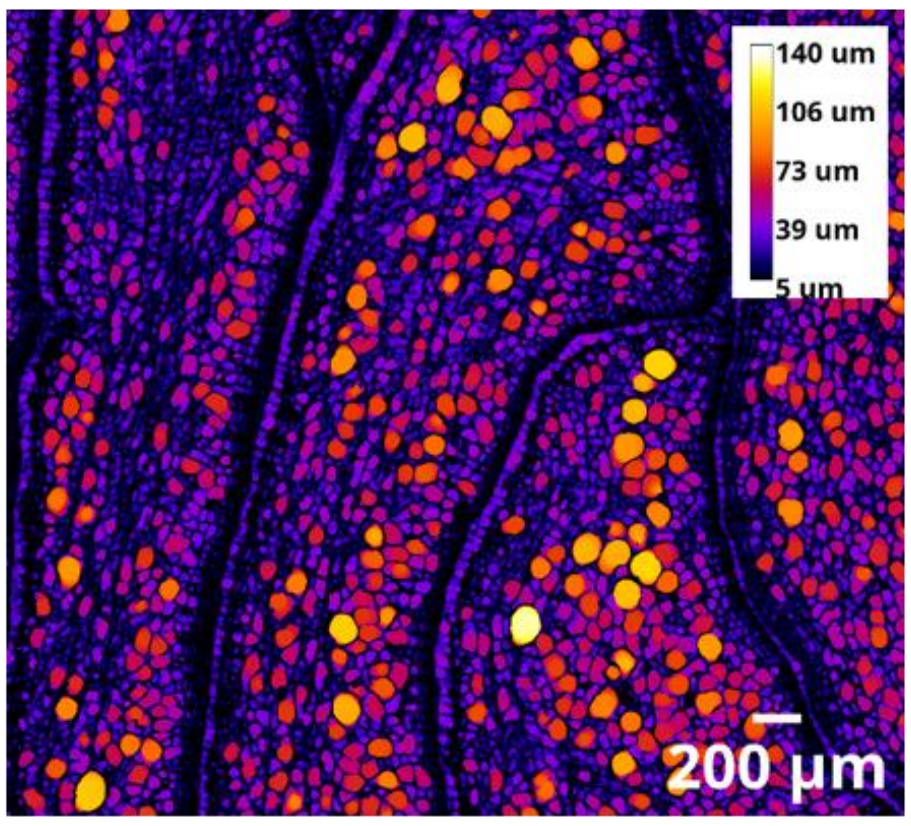

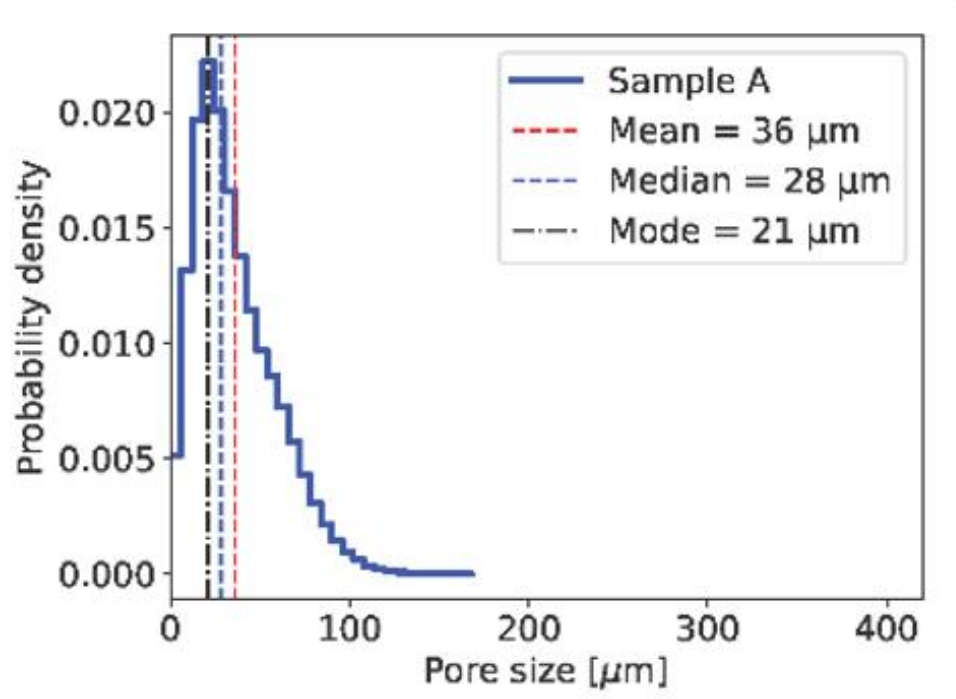

Control

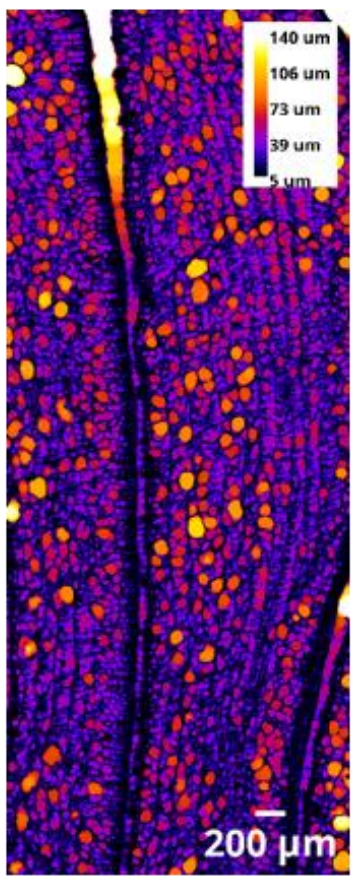

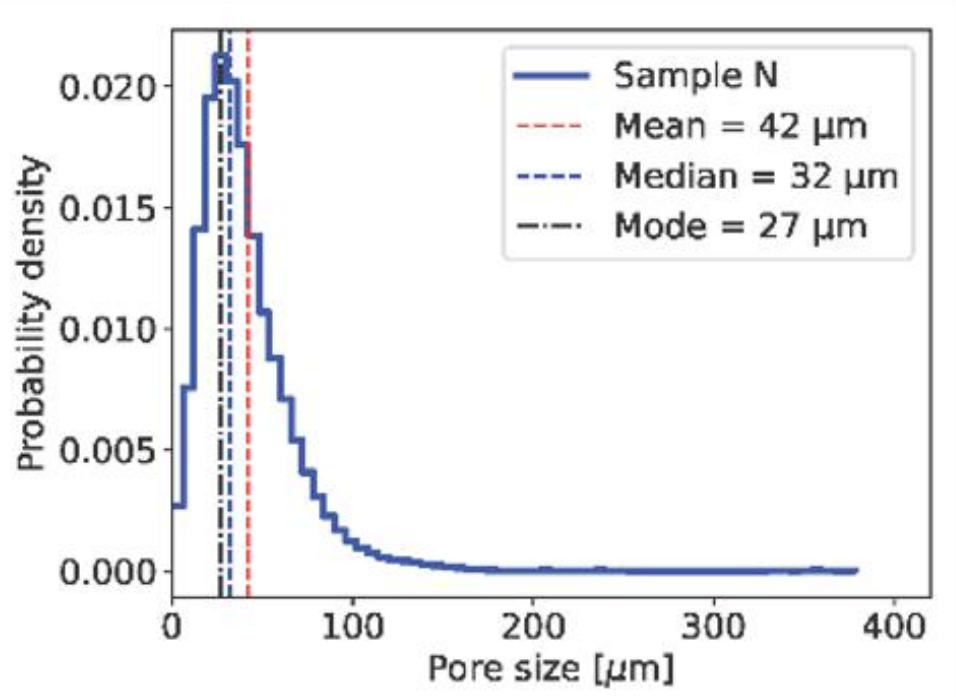

All extractives removed

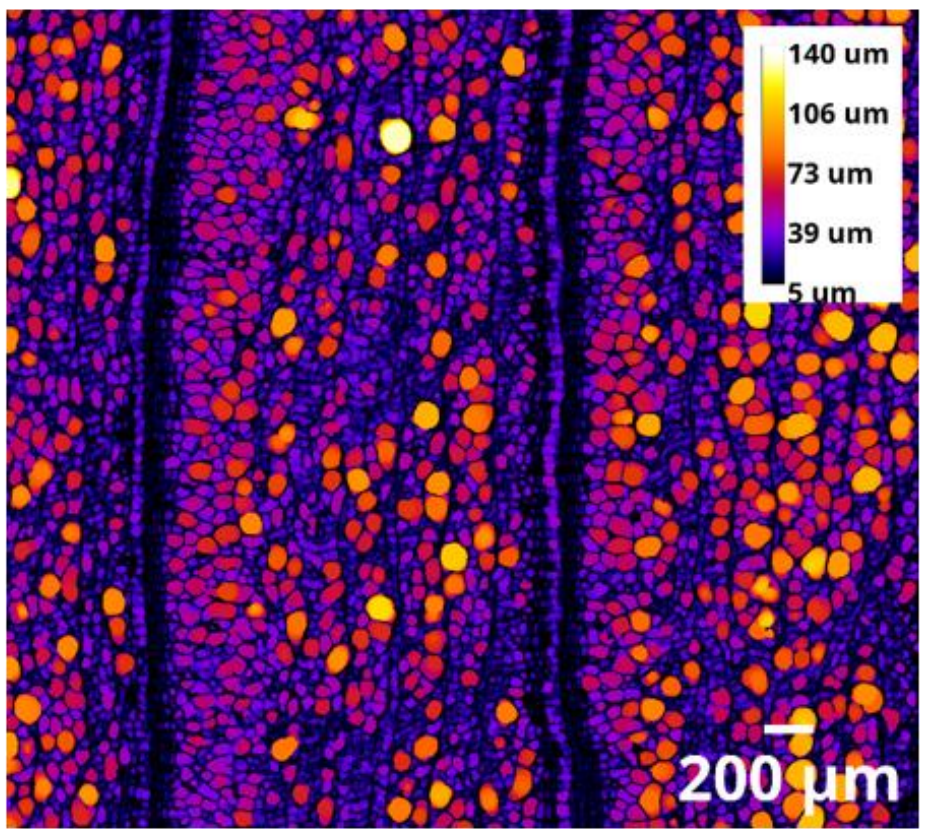

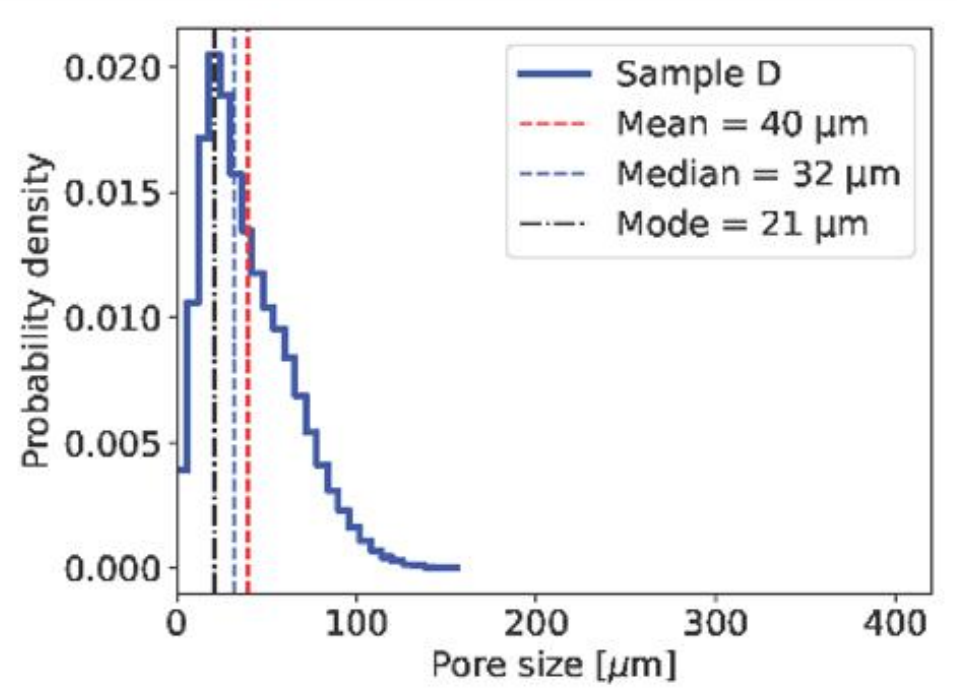

Lipophilic fraction removed

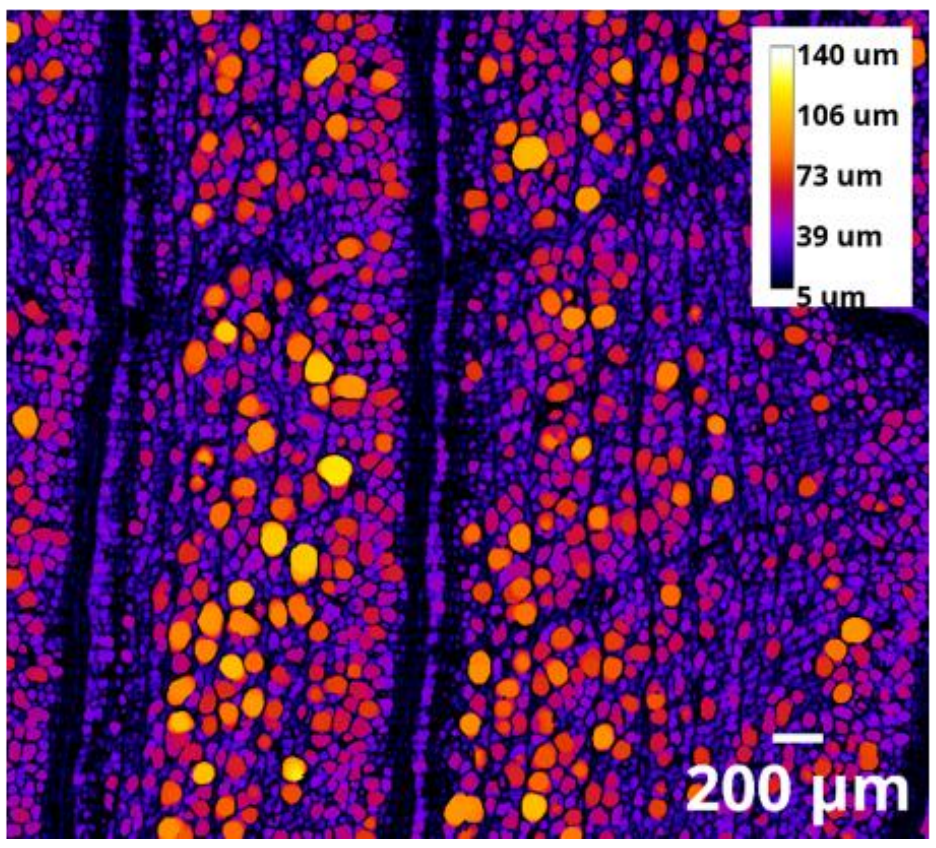

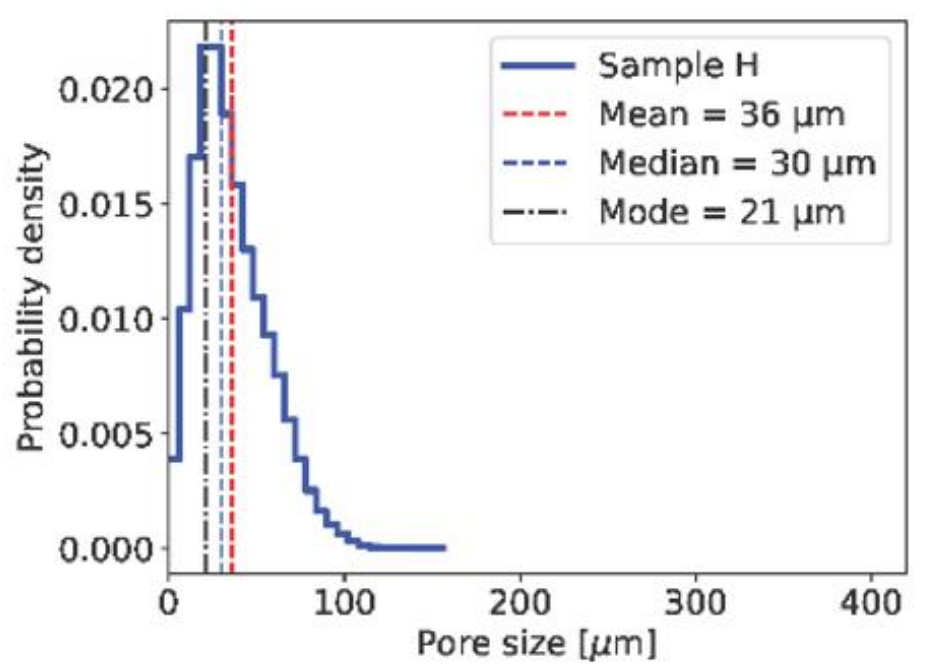

Hydrophilic fraction removed (Soxhlet)

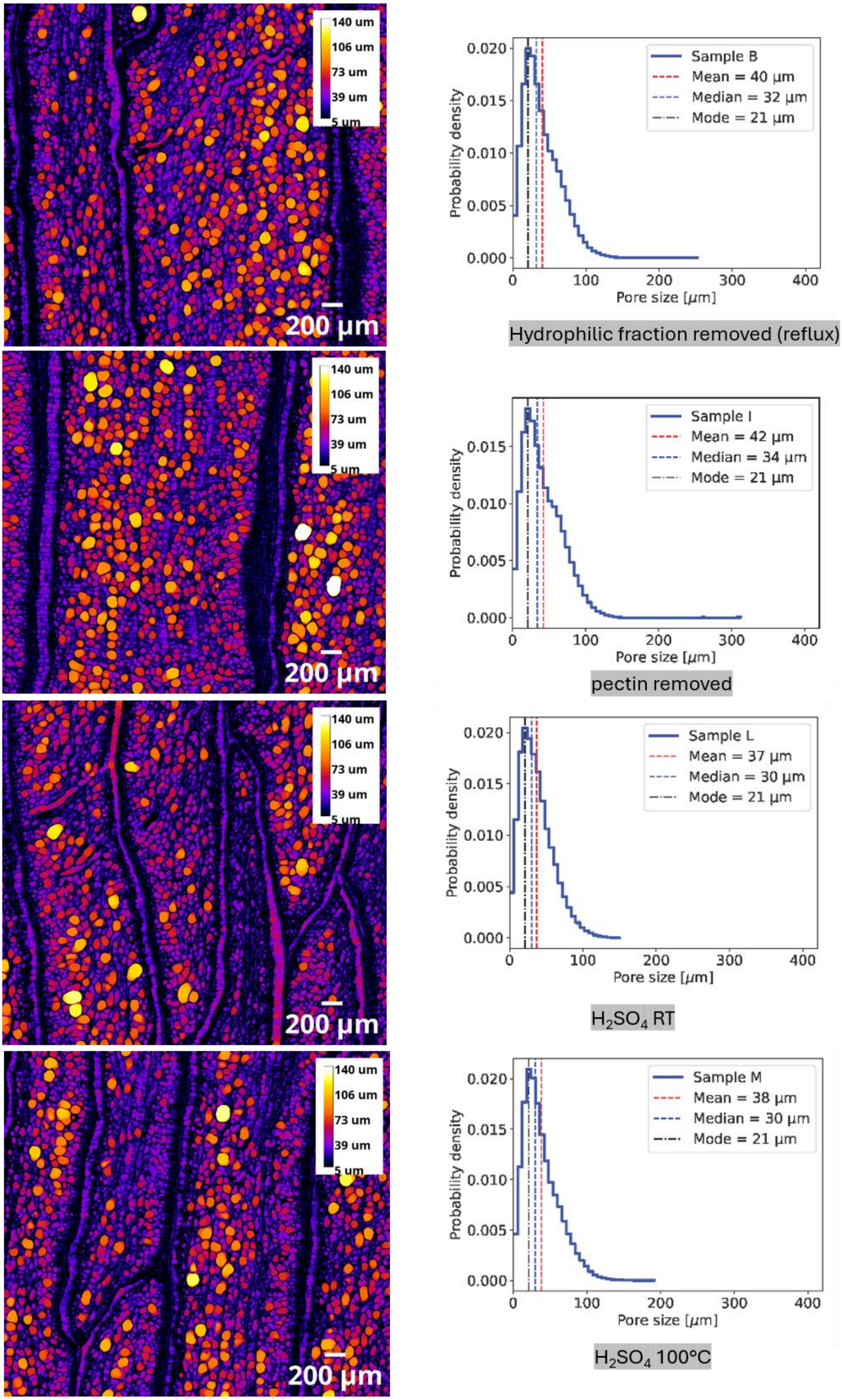
140 um
106 um
73 um
39 um
5 um
200 µm
Sample B
Mean = 40 µm
Median = 32 µm
Mode = 21 µm
Probability density
Pore size [µm]
Hydrophilic fraction removed (reflux)
Sample I
Mean = 42 µm
Median = 34 µm
Mode = 21 µm
pectin removed
Sample L
Mean = 37 µm
Median = 30 µm
Mode = 21 µm
H_2SO_4 RT
Sample M
Mean = 38 µm
Median = 30 µm
Mode = 21 µm
H_2SO_4 100°C

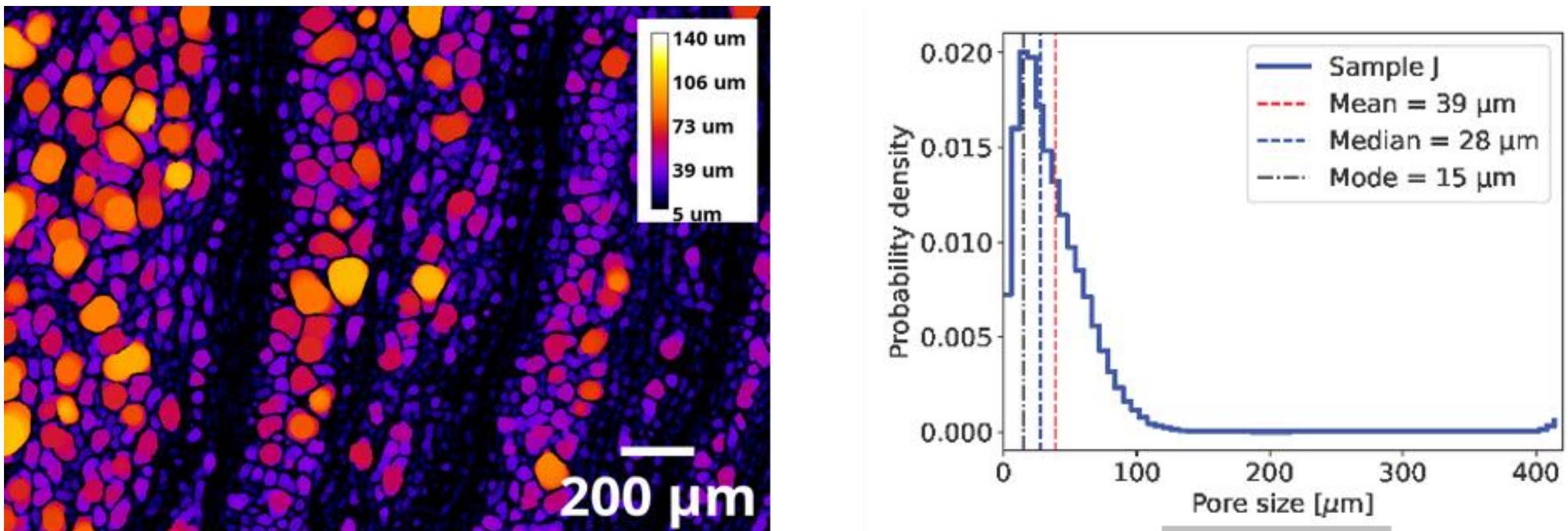

NaOH 1% RT

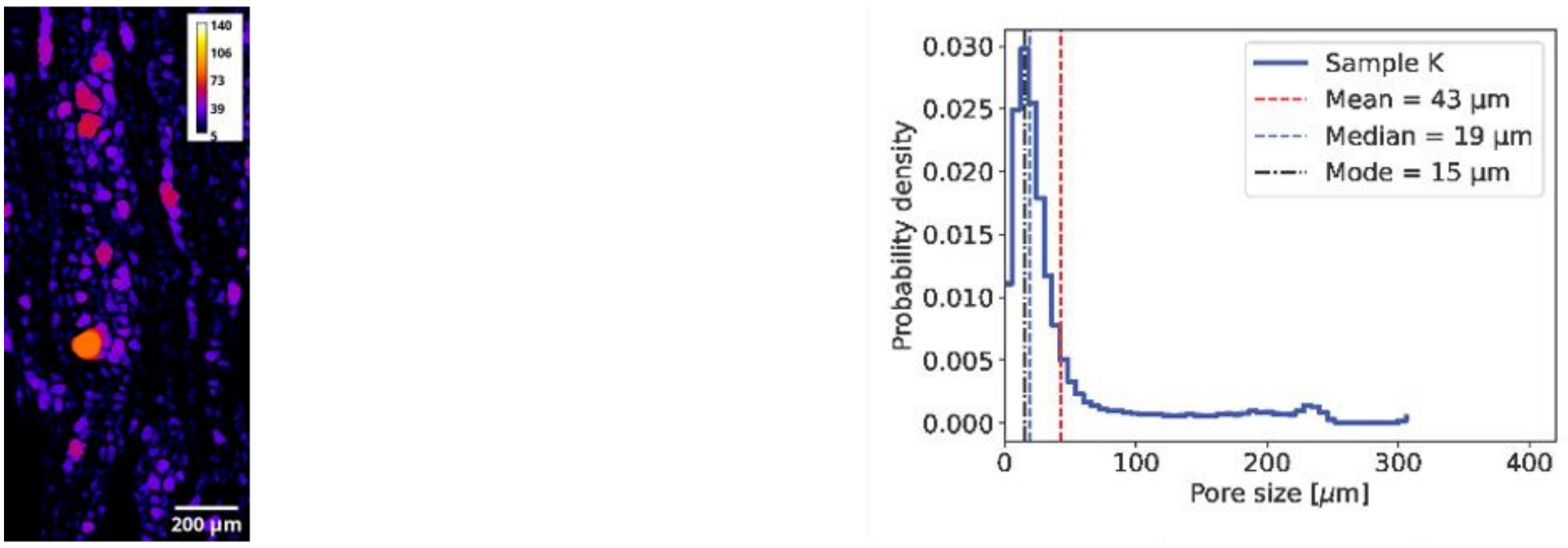

NaOH 1% 100°C

Figure S7. Pore size histograms: Control, All extractives removed, Lipophilic (hexane soluble) fraction removed, Hydrophilic fraction removed (Soxhlet), Hydrophilic fraction removed (reflux), pectin removed, H2SO4 RT, H2SO4 100°C, NaOH 1% RT treatment, NaOH 1% 100°C treatment.

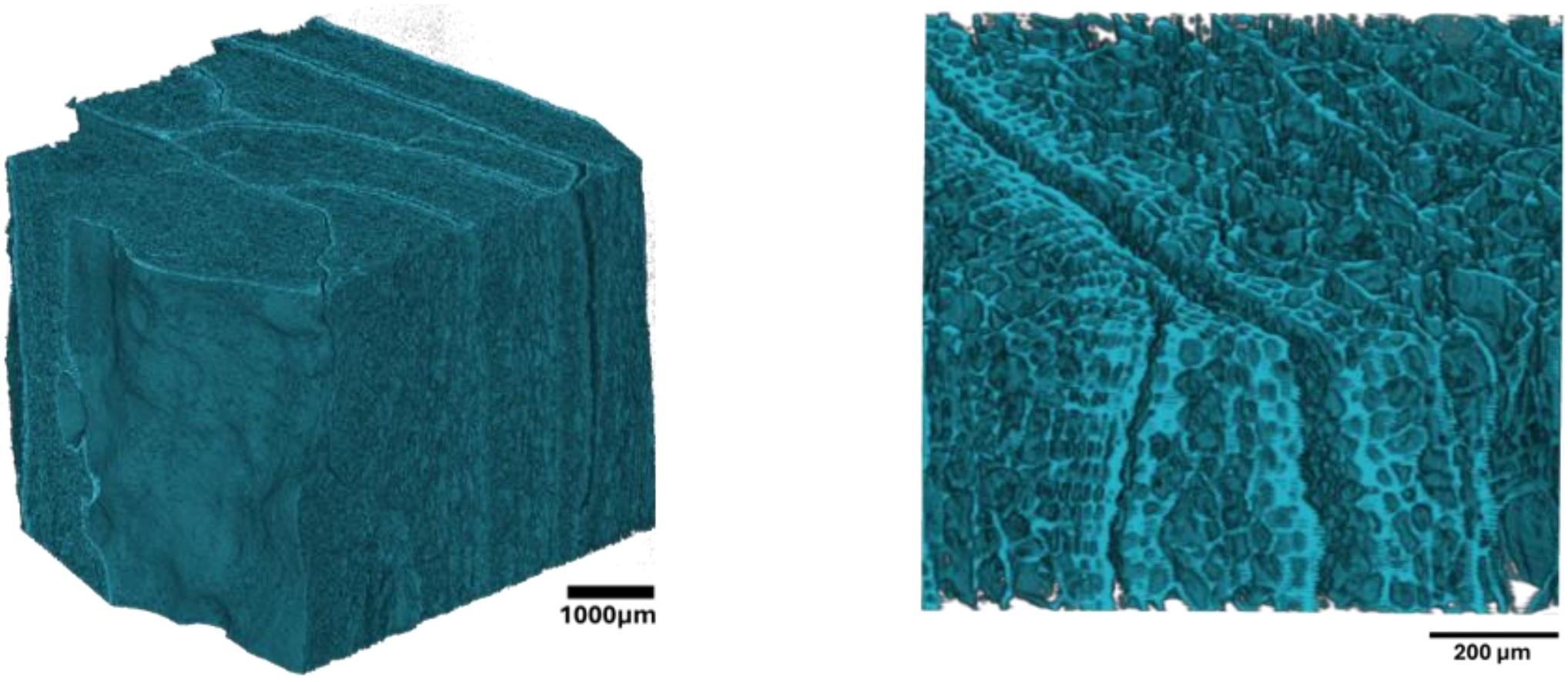

Control

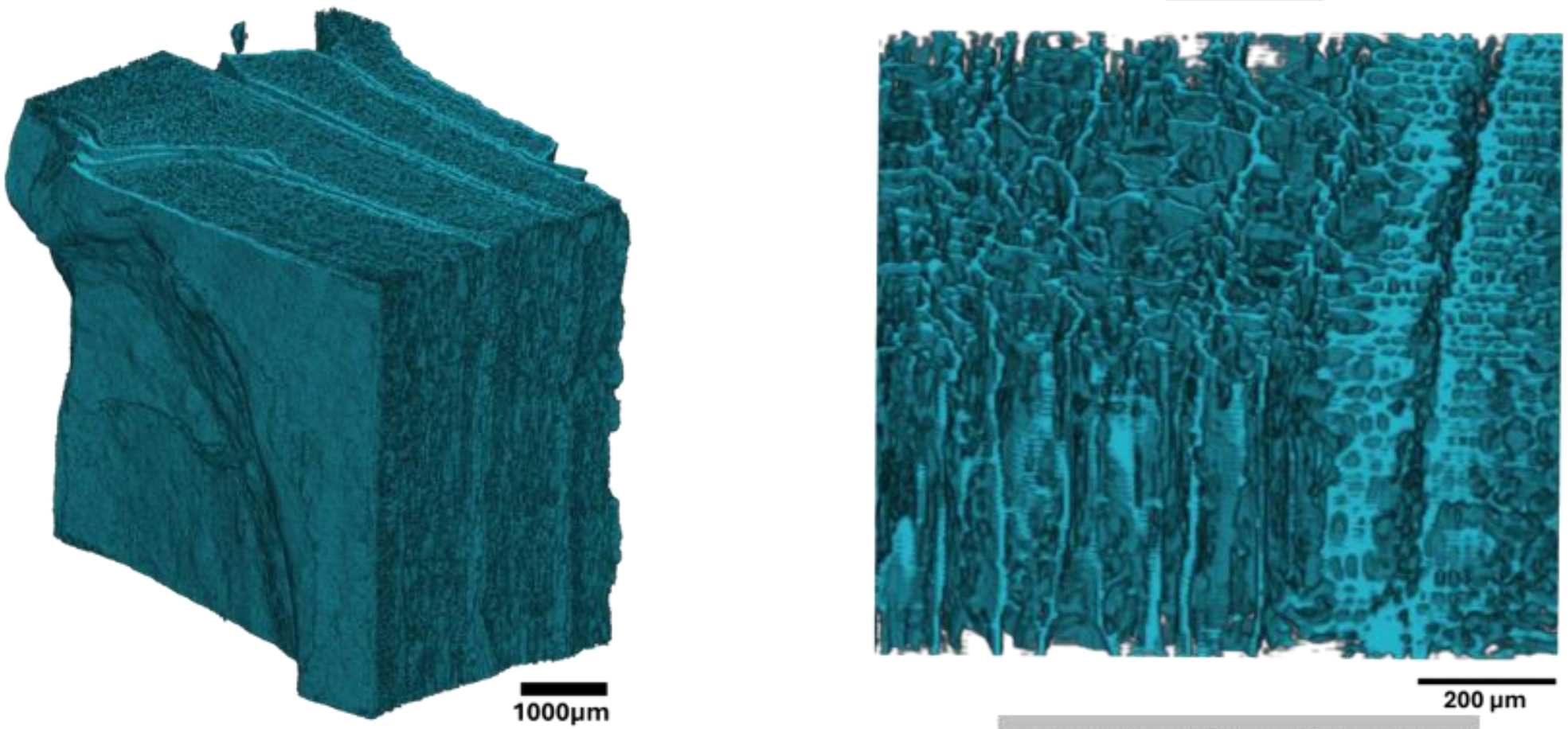

All extractives removed

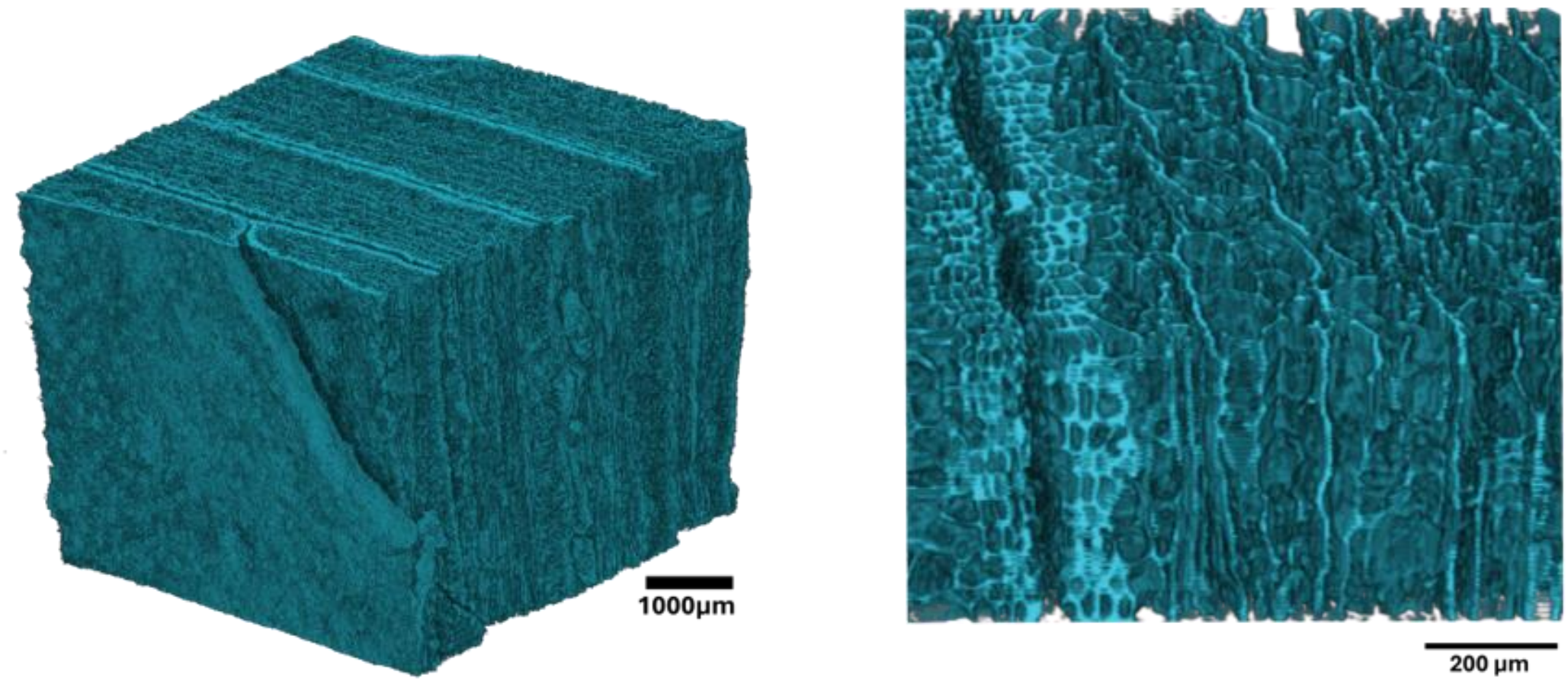

Lipophilic fraction removed

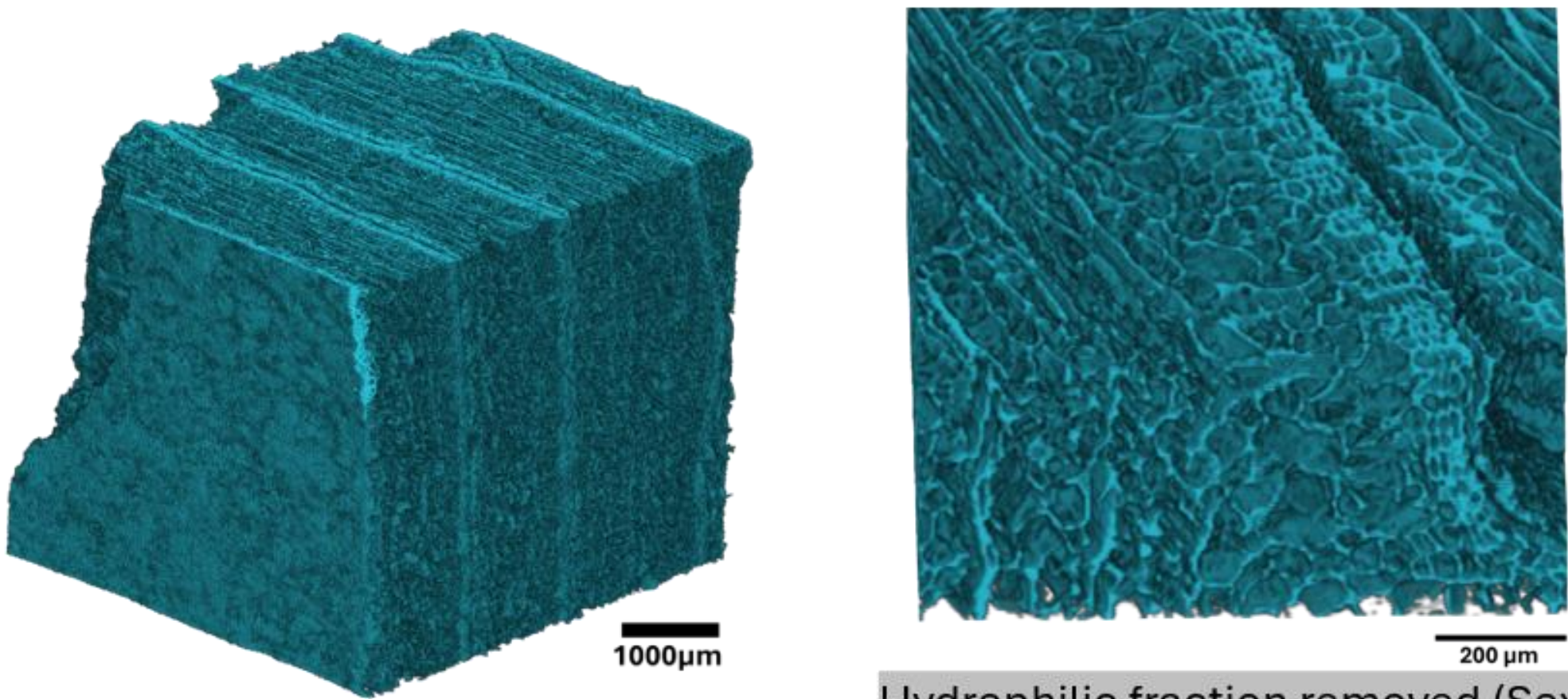

Hydrophilic fraction removed (Soxhlet)

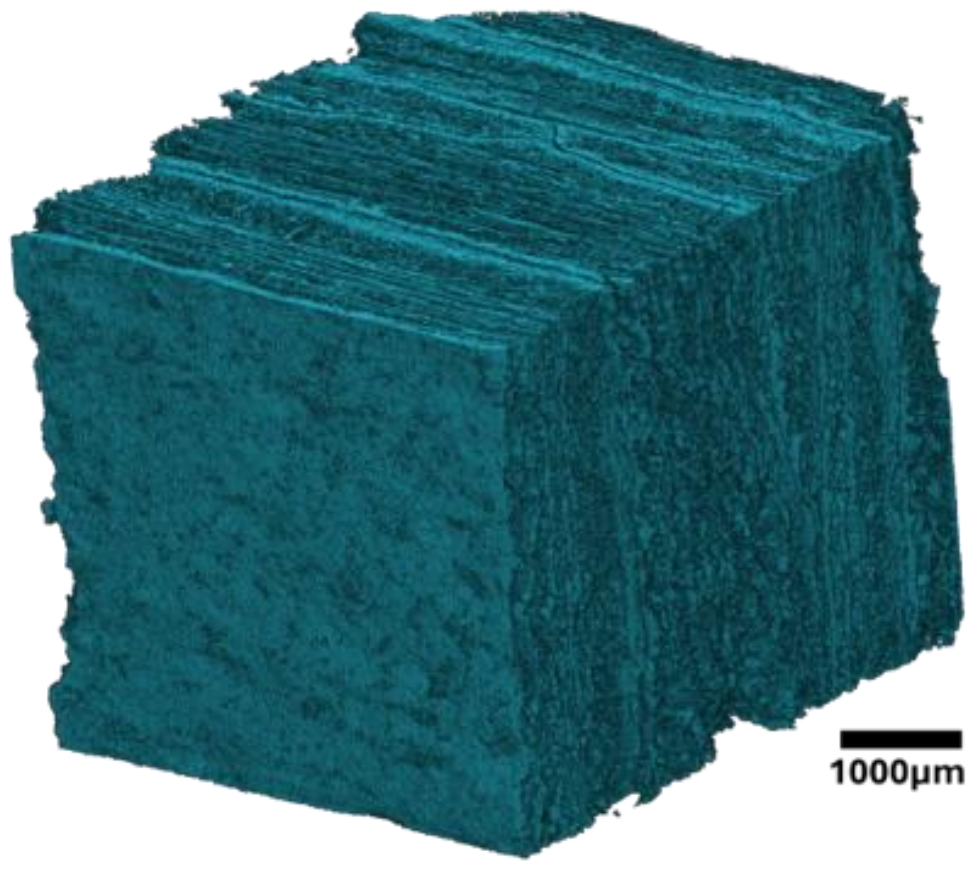

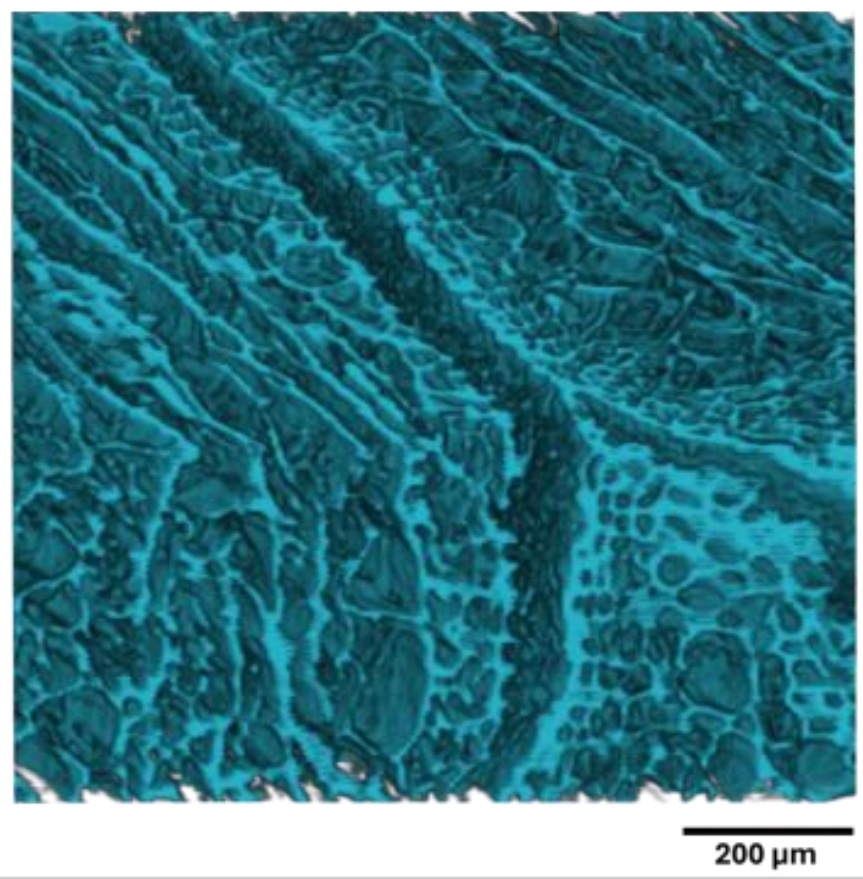

Hydrophilic fraction removed (reflux)

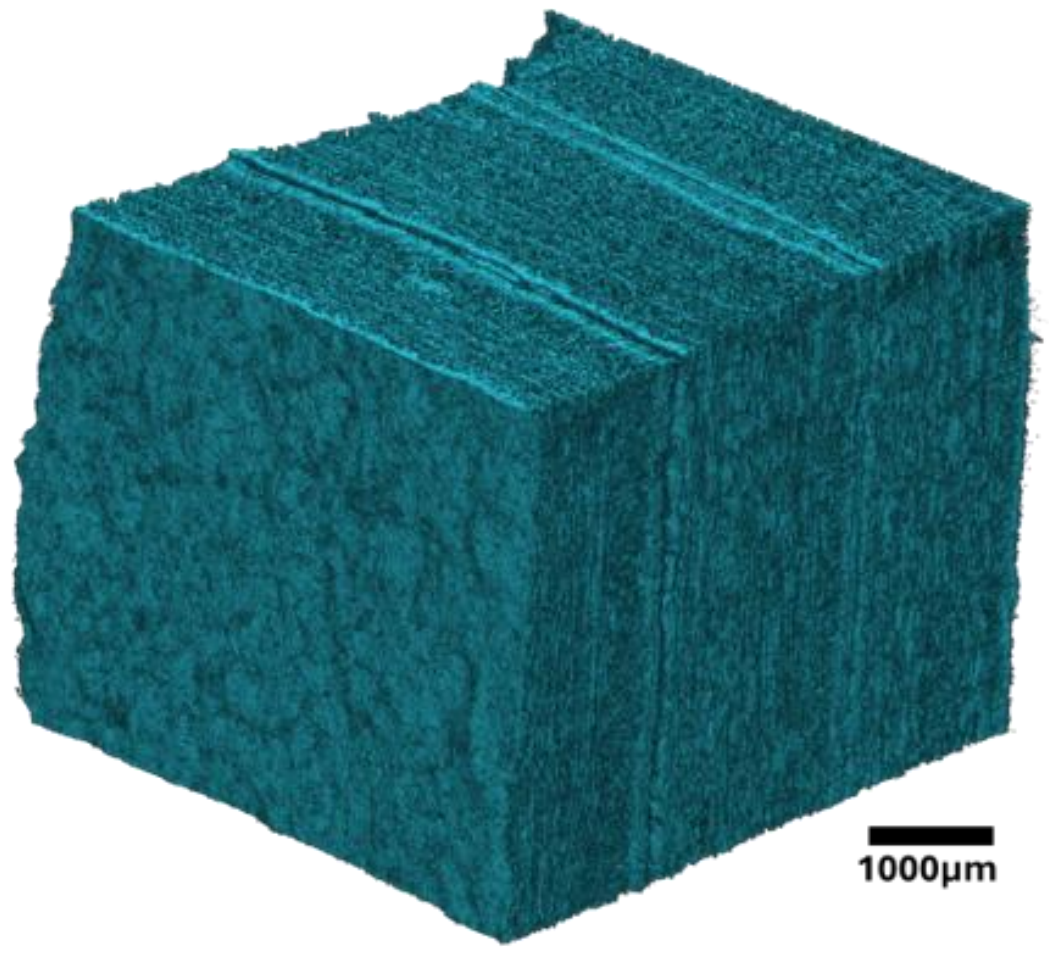

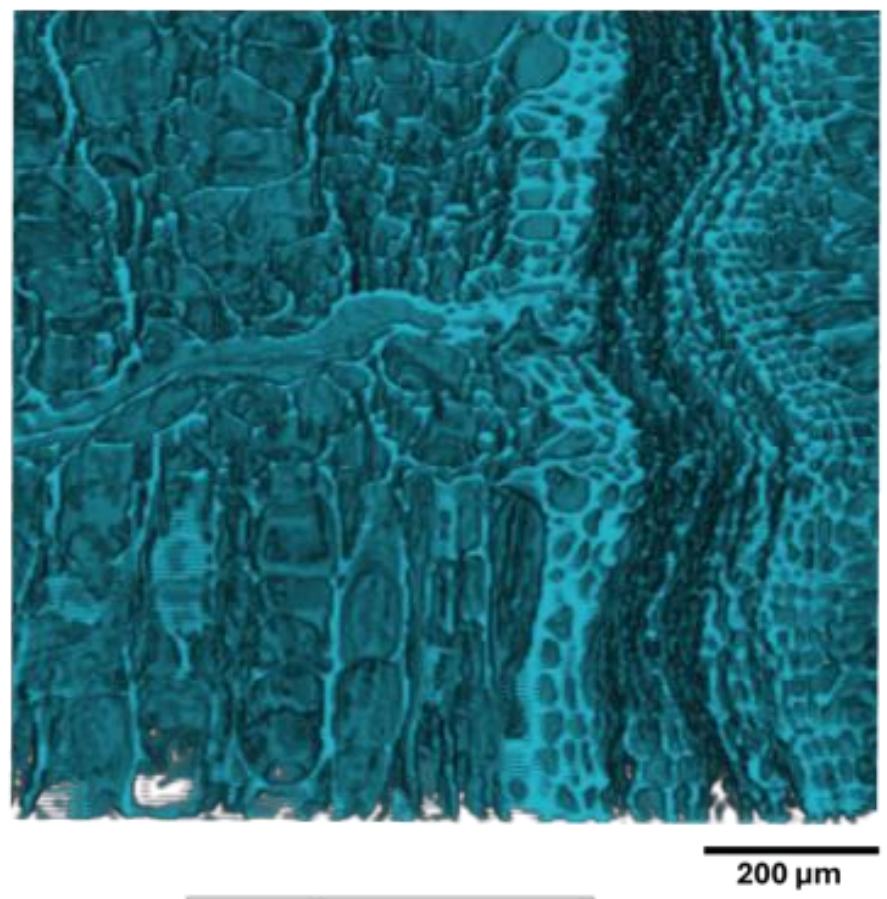

pectin removed

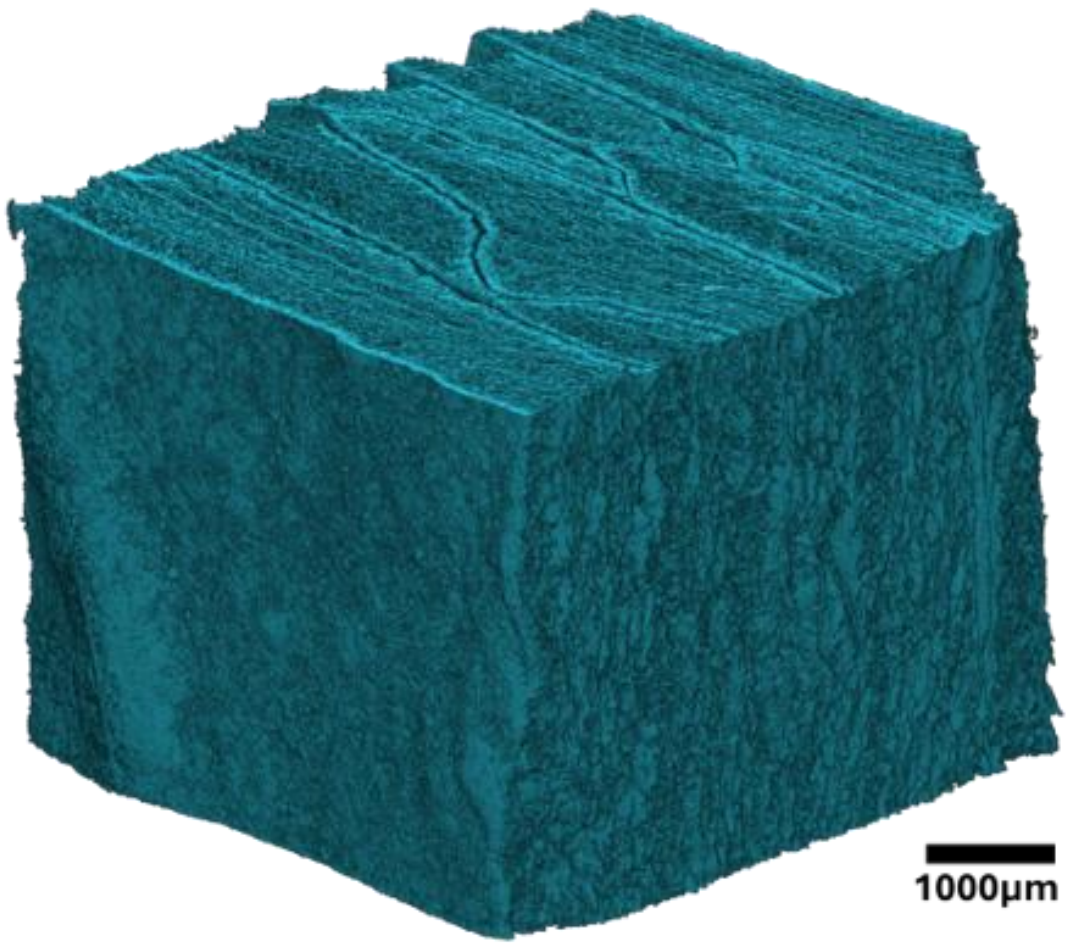

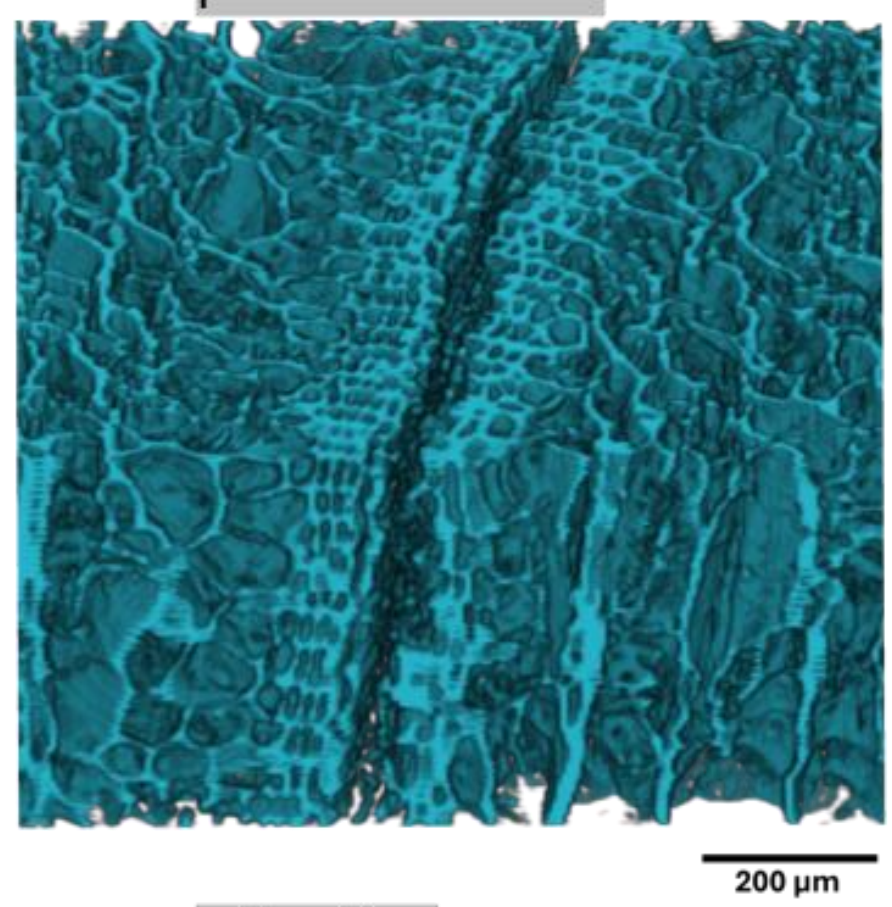

H_2SO_4 RT

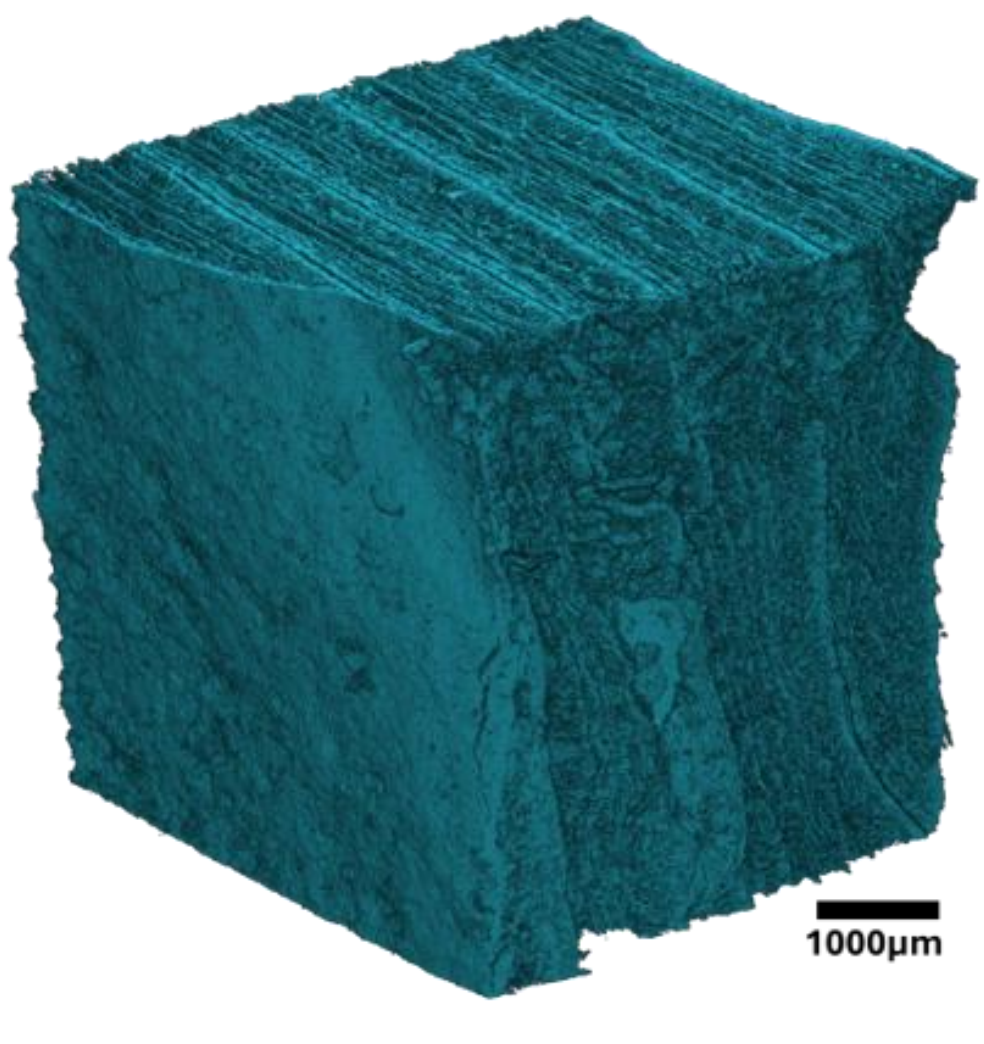

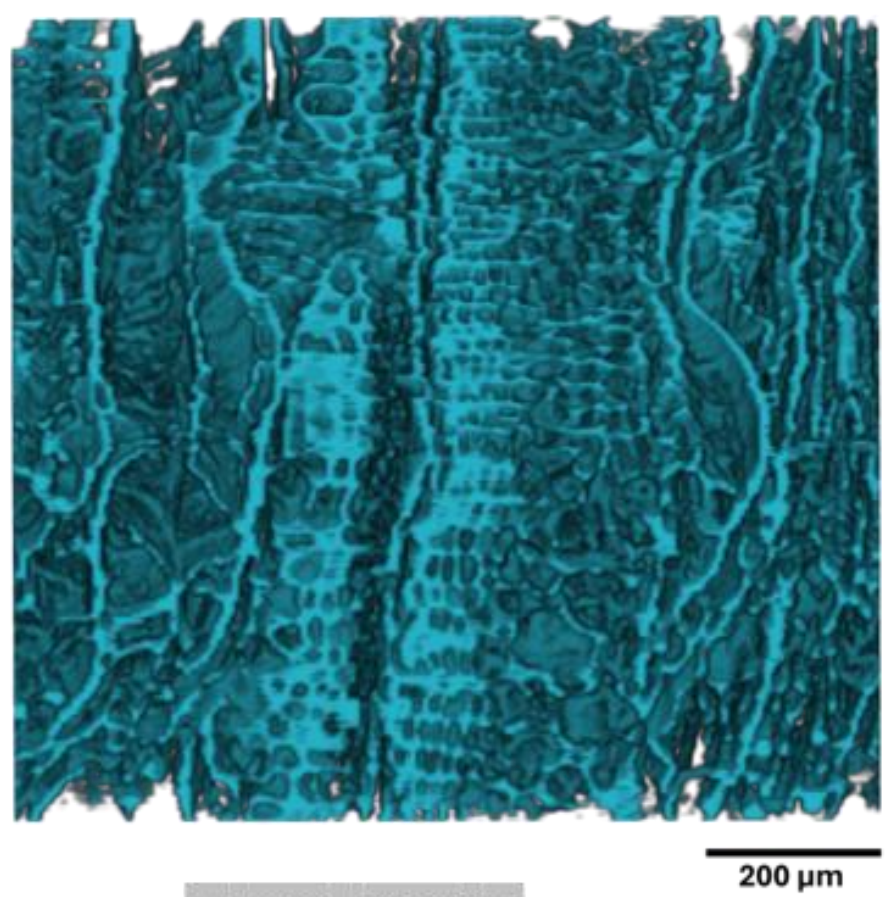

H_2SO_4 100°C

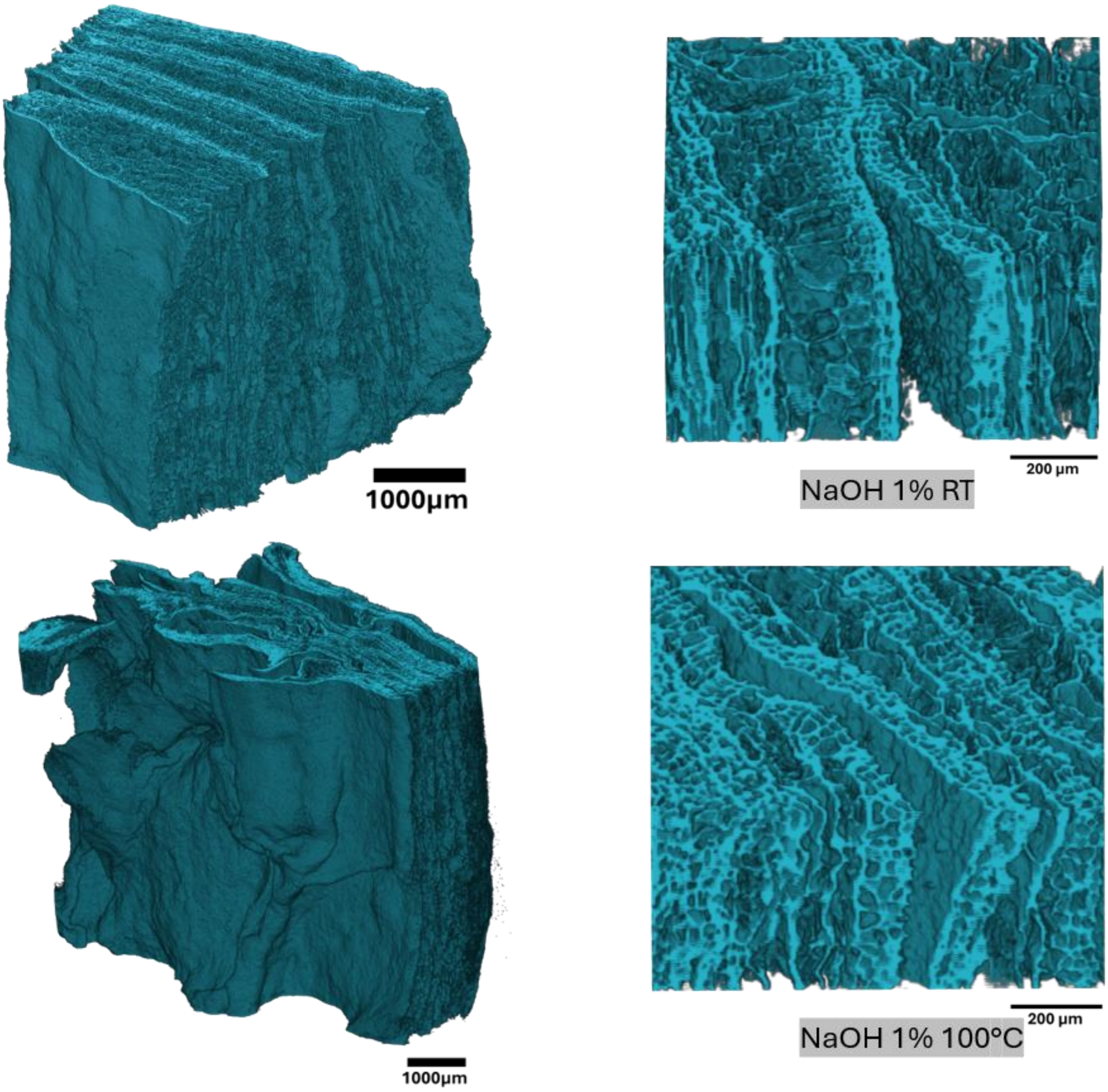

Figure S8. 3D tomographic visuals: Control, All extractives removed, Lipophilic (hexane soluble) fraction removed, Hydrophilic fraction removed (Soxhlet), Hydrophilic fraction removed (reflux), pectin removed, H_2SO4 RT, H_2SO4 100°C, NaOH 1% RT treatment, NaOH 1% 100°C treatment.

Supporting Discussion: GC-MS of extractives

Major lipophilic groups detected in hexane soluble extractives are steroids (cholest-4-en-3-one, stigmast-5-ene), fatty acids (behenic acid, lignoceric acid, arachidic acid, oleic acid, palmitic acid, stearic acid), resin acids (dehydroabietic acid, pimaric acid), terpenoids, terpenes (betulin, isopimaric acid), monoacylglycerols (1-monopalmitin, glycerol monostearate, 2,3-dihydroxypropyl icosanoate).

Water soluble extractives detected by GC-MS are monosaccharaides (d-ribose, arabinofuranose, d-allopyranose, l-rhamnose, galactopyranose, d-glucopyranose, xylopyranose), sugar alcohols (ribitol, d-mannitol, xylitol), flavonoids (taxifolin), carboxylic acids (lactic acid, citric acid), phenolic carboxylic acids (salicylic acid).

Acetone extractives composition indicates less selective extraction: steroids and sterols, triterpenoids, fatty components such as long￼chain saturated fatty acids (palmitic, stearic, arachidic, behenic, lignoceric) and their esters/amides, aliphatic dicarboxylic acids (dodecanedioic acid), and monoacylglycerols and long￼chain fatty alcohols. Also well represented are diterpenoids, and a set of phenolic/phenylpropanoid compounds (ferulic and protocatechuic acids, phenols). Less abundant classes include flavonoids (taxifolin), sugar or polyol derivatives (glucose, mannitol), sesquiterpenoids/terpenoid alcohols (bicyclic diterpenoid alcohols).

Ethanol extractives include diterpenoids, triterpenoids (lupane-type betulin and lup-20(29)-ene derivatives, and cycloartane/lanostane-type triterpenes). Fatty components, including long-chain saturated fatty acids (palmitic, stearic, arachidic, behenic, lignoceric and branched isoprenoid C16) and their esters, long-chain fatty alcohols and diols, monoacylglycerols, and dicarboxylic acids (dodecanedioic and 10,12-docosadiynedioic). Also abundant are steroids/sterols (cholestane- and stigmasterol-type, bile acids and pregnane/androstane derivatives, mostly silylated), and phenolic/phenylpropanoid compounds (BHA and other hindered phenols, protocatechuic and related acids, ferulate/p-coumarate wax esters, nortotarol-type phenols). Less common but present are flavonoids (taxifolin), small carbohydrate derivatives (silylated glucose, mannitol, arabitol, pinitol), various terpenoid hydrocarbons and monoterpenoids/sesquiterpenoids, plus minor xanthine alkaloids (theophylline), simple amides and derivatization reagents (e.g. N-(dimethylamino)methylene-2,2,2-trifluoroacetamide), which together make up the lower-abundance tail of the chromatographic profile.

Band (cm^{-1})	Vibration	Components
854-858	α-glycosidic linkage skeletal vibration	pectin [1,2,3]
1092-1096	C-O-C glycosidic bond stretching	cellulose [1,3]
1600-1608	Aromatic ring stretching	lignin [1,3], phenolic extractives*[2,3]
1620	Conjugated C=C stretching	lignin [1,3], phenolic extractives*[2,3]
1634	Conjugated C=C stretching	lignin [1,3], phenolic extractives*[2,3]
1654-1658	Conjugated C=C or C=O stretching	lignin, lipophilic extractives*[1,2,3]
1666	Conjugated C=O stretching (α,β-unsaturated ketone structures)	lignin/ extractives [3]
1684	Conjugated C=O stretching (α,β-unsaturated ketone structures)	lignin/ extractives [3]
2900-2910	Aliphatic CH_2 symmetric stretching	cellulose [1,3], lipophilic extractives [1,3]
2940-2945	Aliphatic CH_3/CH_2 asymmetric stretching	lignin (methoxy groups) [1,3], pectin [1,2,3], lipophilic extractives [1,3]

Table S1. Interpretation of Raman bands. Given bands are seen in reference compounds from the samples in this work, and discussed in more detail in previous works (14, 37, 38).

** Examples of lipophilic extractives common for pine are abietic, isopimaric, linoleic, oleic acids, also detected in GC-MS in this study. Phenolic compounds include lignin polyphenolic structures and small phenolics as stilbenes and pinosylvins.*

1 = observed from pure compound spectra in this study, 2 = observed from spectra of component-removed samples, 3 = discussed in previous studies.

Hexane extractives		
Compound Name	Retention Time (min)	Area %
Cholest-4-en-3-one	61.6	8.9
Stigmast-5-ene, 3β-(trimethylsiloxy)-, (24S)-	60	8.5
Dehydroabietic acid, TMS derivative	43.6	7.8
Behenic acid, TMS derivative	48.4	6.9
C(14a)-Homo-27-norgammacer-13-en-21-ol, 3-methoxy-, (3α,21β)-	67.2	4.6

trimethyl((2-((2S,4aS)-4a-methyl-8-methylenedecahydronaphthalen-2-yl)propan-2-yl)oxy)silane	68.2	4.5
Lignoceric acid, TMS derivative	51.9	3.9
1-Monopalmitin, 2TMS derivative	47.6	3.6
Pimaric acid, TMS derivative	41.8	2.5
Arachidic acid, TMS derivative	44.6	2
Oleic Acid, (Z)-, TMS derivative	39.9	2
Glycerol monostearate, 2TMS derivative	51.1	1.9
2,3-Bis((trimethylsilyl)oxy)propyl docosanoate	57.5	1.8
((2-((2R,4aR)-4a,8-dimethyl-1,2,3,4,4a,5,6,8a-octahydronaphthalen-2-yl)propan-2-yl)oxy)trimethylsilane	71	1.5
Palmitic Acid, TMS derivative	36.1	1.4
2,3-Dihydroxypropyl icosanoate, 2TMS derivative	60.4	1.4
Stearic acid, TMS derivative	39.7	1.3
Palmitoleamide	42.9	1.3
Stigmastane-3,6-dione, (5α)-	64.6	1.2
3β,16β,17α-Tris(trimethylsiloxy)androst-5-ene	46.5	1.2
Cholest-5-en-3-ol (3β)-, tetradecanoate	62.1	1.1
Betulin	65.6	1.1
Lup-20(29)-ene-3,21-dione, 28-hydroxy-	69.3	1.1
Isopimaric acid, TMS	42.4	0.9
Lup-20(29)-en-3-ol, acetate, (3β)-	66	0.9
9,19-Cyclocholestene-3,7-diol, 4,14-dimethyl-, 3-acetate	73.4	0.9
N-((Dimethylamino)methylene)-2,2,2-trifluoroacetamide	11.5	0.9
Lup-20(29)-en-28-al, 3-(trimethylsilyl)oxy, (3β)-	66.9	0.8

Lanosta-7,9(11)-dien-18-oic acid, 3,20-dihydroxy-25-methoxy-, γ-lactone, (3β,20ξ)-	61	0.8
9,19-Cyclolanost-23-ene-3,25-diol, (3β,23E)-	77.6	0.7
9,19-Cyclolanost-24-en-3-ol, (3β)-, TMS derivative	72	0.7
Docosanol, TMS derivative	46.6	0.6
Campesterol, TMS derivative	58.6	0.6
Abietic acid, TMS derivative	44.1	0.5
Dodecanedioic acid, 2TMS derivative	55.7	0.5

Water extractives		
Compound Name	Retention Time (min)	Area %
D-Ribose, 4TMS derivative	25.9, 26.8	42.7
Arabinofuranose, 1,2,3,5-tetrakis-O-(trimethylsilyl)-	27.5	5.9
L-(-)-Arabitol, 5TMS derivative	28.4	5
β-D-Allopyranose, 5TMS derivative	33.6	3.8
β-D-Glucopyranose, 5TMS derivative	33.2	2.7
D-(+)-Galactopyranose, 5TMS derivative (isomer 2)	32.5	2.5
D-Glucose, 5TMS derivative	35.5	2.5
L-Rhamnose, 4TMS derivative	26.2	2.5
D-Psicofuranose, pentakis(trimethylsilyl) ether (isomer 2)	30.9	2.5
D-Mannitol, 6TMS derivative	34.2	2.1
Xylitol, 5TMS derivative	28.8	2
Lactose, 8TMS derivative	51.3	1.8

D-Galactose, 5TMS derivative	29.8	1.4
1-Monopalmitin, 2TMS derivative	47.6	1.3
9-Octadecenamide, (Z)-	42.9	1.1
Glycerol monostearate, 2TMS derivative	51.1	1.1
Silane, [bicyclo[4.2.0]octa-3,7-diene-7,8-diylbis(oxy)]bis[trimethyl-	36	1.1
Taxifolin, 5O-TMS	54.7	0.9
Uridine, 3TMS derivative	44.1	0.8
Glycolic acid, 2TMS derivative	7.7	0.8
Glucoheptonolactone, 5TMS derivative	32.8	0.5
Thiodiglycol, 2TMS derivative	8	0.5
Citric acid, 4TMS derivative	31.2	0.4
Lactic Acid, 2TMS derivative	7.2	0.3

Ethanol extractives		
Compound Name	Retention Time (min)	Area %
Dehydroabietic acid, TMS derivative	43.6	11.2
Behenic acid, TMS derivative	48.3	5.9
Stigmast-5-ene, 3β-(trimethylsiloxy)-, (24S)-	60	5.8
7-Oxodehydroabietic acid, trimethylsilyl ester	47.6	5.8
Pimaric acid, TMS derivative	41.8	4.2
Abietic acid, TMS derivative	44.1	3.4
N-((Dimethylamino)methylene)-2,2,2-trifluoroacetamide	11.6	3
Lignoceric acid, TMS derivative	51.8	3
Cholest-4-en-3-one	61.5	2.6
Theophylline, TMS derivative	42.9	2.6
Methyl 2-hydroxyhexadecanoate, TMS derivative	51	2.6
3-tert-Butyl-4-hydroxyanisole, trimethylsilyl ether	46.5	2.2
Octadecane-1,2-diol, 2TMS derivative	46.9	2.1

C(14a)-Homo-27-norgammacer-13-en-21-ol, 3-methoxy-, (3α,21β)-	67.1	1.6
Isopimaric acid, TMS	42.4	1.5
L-(-)-Arabitol, 5TMS derivative	28.8	1.4
2,3-Bis((trimethylsilyl)oxy)propyl docosanoate	57.5	1.4
Taxifolin, 5O-TMS	54.7	1.4
2,4,7,9-Tetramethyl-5-decyne-4,7-diol, 2TMS derivative	36.1	1.3
Hexadecyl-(E)-p-coumarate, trimethylsilyl ether	79.8	1.3
D-Mannitol, 6TMS derivative	34.2	1.3
Glucopyranose, 5TMS derivative	33.2	1.1
Tetracosanol, O-TMS	50.3	1.1
Kaur-16-ene, 18-[(trimethylsilyl)oxy]-, (4α)-	40	1
Deoxycholic Acid, 2TMS derivative	46	1
D-Trehalose, 7TMS derivative	51.3	1
Stigmastane-3,6-dione, (5α)-	64.6	0.9
2,4-Cholestadien-1-one	62.2	0.8
Stearic acid, TMS derivative	40.5	0.8
2-tert-Butyl-4-ethylphenol , trimethylsilyl ether	44.8	0.7
2,3-Dihydroxypropyl icosanoate, 2TMS derivative	60.4	0.7
6H-Chromene-4,5-dione, 2-butyl-3-ethyl-7,7-dimethyl-7,8-dihydro-	65.3	0.7
Protocatechoic acid, 3TMS derivative	30.9	0.6
Silane, [[(3α,5β,20S)-pregn-11-ene-3,11,17,20-tetrayl]tetrakis(oxy)]tetrakis[trimethyl-	48.7	0.6
Terpin, 2TMS derivative	27.8	0.6
Docosanol, TMS derivative	46.7	0.6
trimethyl((2-((2S,4aS)-4a-methyl-8-methylenedecahydronaphthalen-2-yl)propan-2-yl)oxy)silane	67.6	0.6
(1R,4aR,4bS,7R,10aR)-1,4a,7-Trimethyl-7-vinyl-1,2,3,4,4a,4b,5,6,7,9,10,10a-dodecahydrophenanthrene-1-carbaldehyde	38.8	0.5
Lup-20(29)-ene-3,21-dione, 28-hydroxy-	69.2	0.5
trimethyl(((7S)-1,1,7-trimethyl-4-methylenedecahydro-1H-cyclopropa[e]azulen-7-yl)oxy)silane	38.4	0.5
3-O-Acetyl-6-methoxy-cycloartenol	67.3	0.5
Dodecanedioic acid, 2TMS derivative	58.6	0.5
Andrographolide, tri-(trimethylsilyl)-	50.8	0.4
Acetamide, TMS derivative	8	0.4
Bicyclo[5.3.0]decan-2-one, 9-methylene-10-trimethylsilyl-	45.1	0.4

Arachidic acid, TMS derivative	44.4	0.4
11-Ketoetiocholanolone, TMS derivative	47.8	0.4
(S,E)-8,12,15,15-Tetramethyl-4-methylenebicyclo[9.3.1]pentadeca-7,11-diene	37.8	0.4
Betulin	75.8	0.4
Hexadecanoic acid, butyl ester	39.2	0.4
22,23-Dibromostigmasterol acetate	73.3	0.4
10,12-Docosadiynedioic acid, 2TMS derivative	40.3	0.4

Acetone extractives		
Compound Name	Retention Time (min)	Area %
Dehydroabietic acid, TMS derivative	43.6	10.2
Behenic acid, TMS derivative	48.4	6.1
Methandriol, 2TMS derivative	47.5	5.8
β-Sitosterol, TMS derivative	60	5.6
5β-Cholanic acid-3α-[(trimethylsilyl)oxy]-12-one, trimethylsilyl ester	41.8	3.2
Aconitic acid, 3TBDMS derivative	51	2.9
Lignoceric acid, TMS derivative	51.8	2.9
Cholest-4-en-3-one	61.5	2.9
3-tert-Butyl-4-hydroxyanisole, trimethylsilyl ether	46.5	2.9
N-((Dimethylamino)methylene)-2,2,2-trifluoroacetamide	11.6	2.5
Methyl abietate	44.1	2.5
9-Octadecenamide, (Z)-	42.9	2.3
5β-Cholestane-3α,7α,12α,24α,25-pentol TMS	68.1	2.2
Lup-20(29)-en-3-ol, acetate, (3β)-	67.1	1.8
Arachidic acid, TMS derivative	44.6	1.6

Taxifolin, 5O-TMS	54.7	1.5
Tris(2,4-di-tert-butylphenyl) phosphate	64.6	1.5
Androstane-3,17-diol, (3α,5β,17β)-, 2TMS derivative	42.4	1.4
Palmitic Acid, TMS derivative	36.1	1.2
18-O-Feruloyloxyoctadec-9-enoic acid, methyl ester, trimethylsilyl ether	79.8	1.2
Homogentisic acid, 3TMS derivative	49.1	1.2
Cholestan-7-ol, 8,14-epoxy-3-(phenylmethoxy)-, (3β,5α,7α,8α)-	46.9	1.1
Tetracosanol, O-TMS	50.3	1
3,5-Diisopropoxy-1,1,1,7,7,7-hexamethyl-3,5-bis(trimethylsiloxy)tetrasiloxane	47	1
Bicyclo[9.3.1]pentadeca-3,7-dien-12-ol, 4,8,12,15,15-pentamethyl-, [1R-(1R*,3E,7E,11R*,12R*)]-	40	1
Cholesterol, TMS derivative	62.1	0.9
Glucopyranose, 5TMS derivative	33.2	0.9
2,3-Bis((trimethylsilyl)oxy)propyl docosanoate	57.4	0.9
10,12-Docosadiynedioic acid, 2TMS derivative	42.1	0.8
Stearic acid, TMS derivative	40.5	0.7
Betulin	65.6	0.7
Silane, [[(3α,5β,20S)-pregn-11-ene-3,11,17,20-tetrayl]tetrakis(oxy)]tetrakis[trimethyl-	48.7	0.7
25-Hydroxycholesterol, 2TMS derivative	67.7	0.7
1-Naphthalenepropanol, α-ethenyldecahydro-5-(hydroxymethyl)-α,5,8a-trimethyl-2-methylene-, [1S-[1α(S*),4aβ,8aα]]-	69.2	0.6
1-Octacosanol, TMS derivative	46.7	0.6
2,3-Dihydroxy-2-methylpropanoic acid, 3TMS derivative	40.2	0.6
2,3-Dihydroxypropyl icosanoate, 2TMS derivative	60.4	0.6

Hexadecanoic acid, butyl ester	39.2	0.5
(+)-Prostaglandin F2α, 4TMS derivative	50.5	0.5
α-Hydroxyisobutyric acid, 2TMS derivative	49.9	0.5
25,25-Dinor-9,19-cyclolanost-24-yne-, 3-(t-butyldimethylsilyloxy)-	73.3	0.5
Phenol, 2,4,6-tri-tert-butyl-	65.3	0.5
Dodecanedioic acid, 2TMS derivative	58.6	0.4

Table S2. Compounds identified via GCMSsolution and LabSolutions software with the NIST 2017 (National Institute of Standards and Technology) GC-MS mass spectral library. Qualitative and semi-quantitative data reported with compounds of a similarity rate over 90%.
